# Immunosenescence Study of T Cells: A Systematic Review

**DOI:** 10.3389/fimmu.2020.604591

**Published:** 2021-01-15

**Authors:** Ivon Johanna Rodriguez, Nicolás Lalinde Ruiz, Manuela Llano León, Laura Martínez Enríquez, María del Pilar Montilla Velásquez, Juan Pablo Ortiz Aguirre, Oscar Mauricio Rodríguez Bohórquez, Esteban Alejandro Velandia Vargas, Edgar Debray Hernández, Carlos Alberto Parra López

**Affiliations:** ^1^ Laboratorio de Inmunología y medicina traslacional, Departamento de Microbiología, Universidad Nacional de Colombia, Bogotá, Colombia; ^2^ Departamento de Movimiento Corporal Humano, Universidad Nacional de Colombia, Bogotá, Colombia

**Keywords:** cytokines, T cells, immunosenescence, immunosenescence markers, flow cytometry

## Abstract

**Background:**

Aging is accompanied by alterations in immune response which leads to increased susceptibility to infectious diseases, cancer, autoimmunity, and inflammatory disorders. This decline in immune function is termed as immunosenescence; however, the mechanisms are not fully elucidated. Experimental approaches of adaptive immunity, particularly for T cells, have been the main focus of immunosenescence research. This systematic review evaluates and discusses T cell markers implicated in immunosenescence.

**Objective:**

To determine the best flow cytometry markers of circulating T cells associated with immunosenescence.

**Methods:**

We systematically queried PubMed, MEDLINE, EBSCO, and BVS databases for original articles focused on two age groups of healthy humans: 18–44 (young adults) and >60 (older adults) years. In accordance with the Cochrane methodology, we synthesized data through qualitative descriptions and quantitative random effects meta-analysis due to extensive heterogeneity.

**Results:**

A total of 36 studies conducted in the last 20 years were included for the qualitative analysis and four out of these studies were used to perform the meta-analysis. A significant decrease in naïve T cell subset was observed in older adults compared to young adults. Primary markers used to identify senescent cells were loss of CD28 and increased expression of CD57 and KLRG1 in terminally-differentiated memory T cell subset in older adults. Moreover, we observed an increase in proinflammatory cytokines and decrease in telomere length in old adult T cells. It was not possible to perform quantitative synthesis on cell markers, cytokines, and telomere length because of the significant variations between the groups, which is attributed to differences in protocols and unreported measurements, thus generating a high risk of bias.

**Conclusions:**

Heterogeneity among studies in terms of data report, measurement techniques and high risk of bias were major impediments for performing a robust statistical analysis that could aid the identification of eligible flow cytometry markers of immunosenescence phenotype in T cells.

## Introduction

The immune system presents a series of age-associated changes that affects its capacity to respond to new challenges. This cellular state, classically referred to as immunosenescence, increases susceptibility to infectious diseases, cancer, and autoimmunity (1). Immunosenescence is characterized by: (i) decreased response to new invading infectious agents, (ii) unsupported memory T cell response, (iii) increased susceptibility to autoimmune diseases, and (iv) chronic low-grade inflammation “inflammaging” (2, 3).

Most studies have focused on evaluating age-associated changes in T cell populations. These cells undergo continuous remodeling as a result of constant interaction with multiple stressors from the internal and external environments. Consequently, reorganization of the immune system is generated throughout lifetime (4). Given the need to maintain the naïve cell repertoire, T cell population is particularly sensitive, thus responding to both chronic or latent infections and new pathogens through clonal expansion and differentiation to effector subpopulation (1).

One of the major age-associated changes that occur in the immune system is thymic involution, which results in variations in number of naïve T cells; with a most notable decrease in CD8 rather than CD4 T cells, since the latter maintain their populations by homeostatic proliferation. This decrease is accompanied by a decline in T cell receptor (TCR) clonal diversity and increase in memory subpopulation, with accumulation of terminally-differentiated T cells that are either dysfunctional or exhausted (5, 6).

Several studies have demonstrated that terminally-differentiated CD8 T cells are less dependent on TCR activation, but more sensitive to innate signals (7). This convergence of characteristics of innate and adaptive immunity in T cells has been described in CD8 T cells that express CD27 and CD28 membrane receptors and exhibit senescence characteristics that includes: (i) low proliferative activity, (ii) shortening of telomeres, (iii) decreased telomerase activity, (iv) expression of senescence-associated markers (e.g., CD57 and KLRG1) and intracellular molecules (e.g., p38 and γH2AX), (v) expression of NK cell markers including inhibitors (KLRG1 and NKG2A) and activators (NKG2C and NKG2D), and (vi) secretion of large amounts of IFNγ and TNFα (7–10).

Immunosenescence is multifactorial and highly dependent on the environment, antigenic challenges, and epigenetic modifications that are particular to individual experiences (immunobiography) (2, 11). Due to the complex diversity of immune aging, it has been proposed that senescence of circulating T cells can be possibly evaluated through the expression of multiple markers such as CD27, CD28, CD57, KLRG1, CD45 isoforms (RA/RO), and production of proinflammatory molecules including IL-6 and TNFα (12). This systematic review aimed to determine the best markers measured by flow cytometry for identifying immunosenescence phenotype in human T cells.

## Methods

### Study Design and Protocol Registration

Our systematic review considered the PRISMA checklist for reporting and design of systematic reviews. The study protocol was registered in PROSPERO (ID protocol: CRD42020171342) on 05/07/2020.

### Search Strategy

We systematically queried PubMed, MEDLINE, EBSCO, and BVS from 1 January, 2000 to 28 October, 2020. The search strategy included only MeSH and DeCS terms and studies published in English and Spanish (**Table S1**). We also employed the advanced search filters to retrieve only experimental articles on human immune cells.

### Eligibility Criteria

To reduce data variability and make accurate comparisons between articles, only published experimental studies regarding flow cytometry analysis of human T cells were evaluated. Memory subsets, senescence and exhaustion-associated markers, cytokine production, and telomere length were the immunosenescence characteristics of interest.

Two age groups were compared: young adults (18–44 years) and older adults (>60 years). The pediatric population was excluded as a result of the high variability in their humoral and cellular response (13). Individuals aged between 45 and 60 years were considered middle-aged adults, thus representing an intermediate population. Therefore, we excluded this group in order to compare the young and older adults only.

Reviews, studies using animal models, and studies using diseased persons were excluded for this systematic review. Only studies published after 2000 were selected since articles from previous years use different measuring techniques and reagents which made their comparison with recent studies difficult.

### Study Selection and Data Collection Process

Duplicates were excluded and two reviewers independently screened the titles and abstracts of retrieved studies by applying the inclusion and exclusion criteria. Finally, reviewers selected the articles whose full text is to be read and evaluated for data extraction. Any disagreement over the eligibility of particular studies was resolved by consensus.

**Figure 1:**
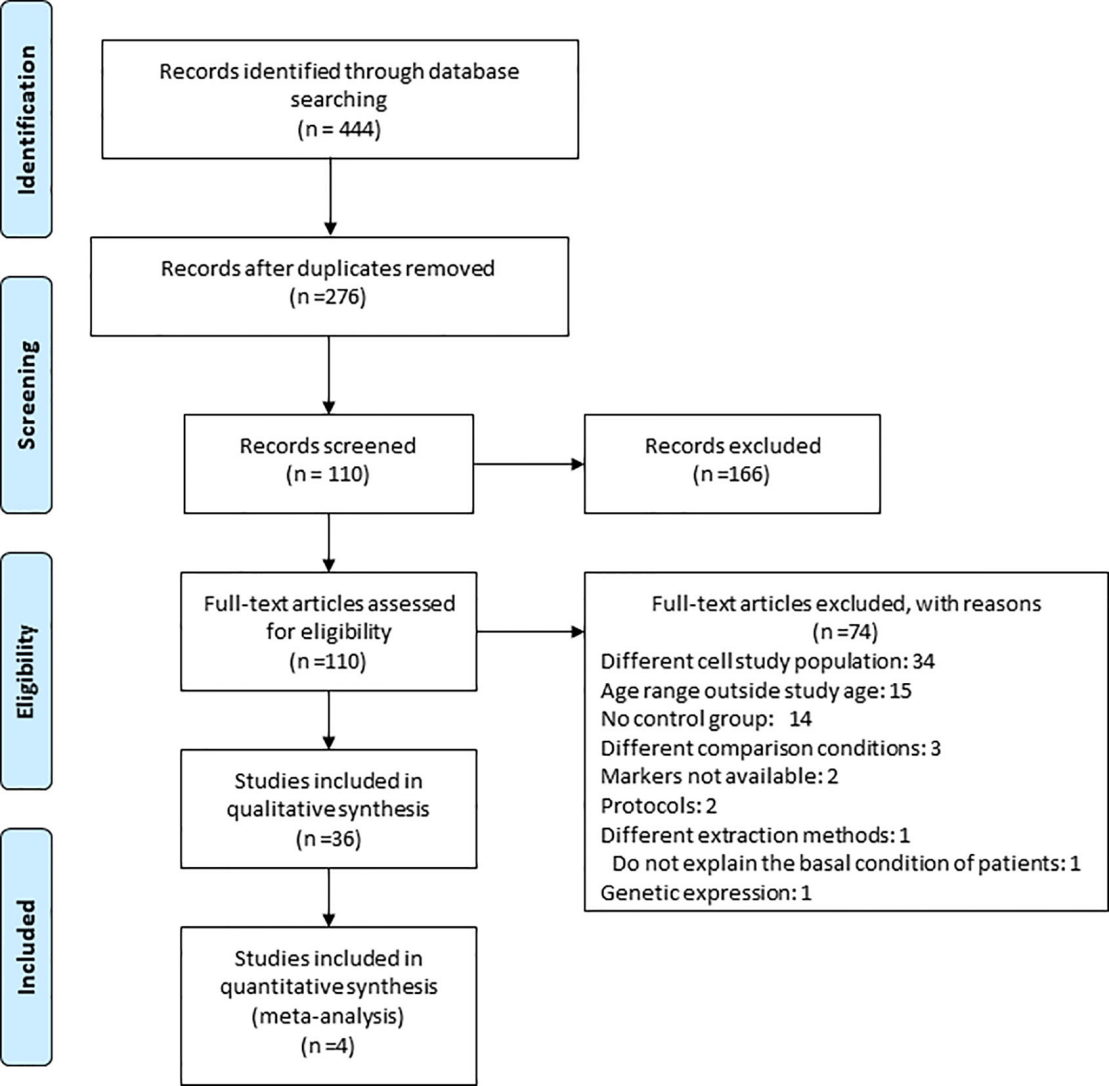
Study flow diagram. The search strategy performed in this article led to the initial selection of 418 potential papers. 303 were retrieved from PUBMED/MEDLINE, 115 from EBSCO and 0 from BVS in the identification phase. After duplicates were removed, a total of 263 papers were identified. From these, 157 articles were excluded based on title and abstract. Further investigation of the remaining 106 full text articles led to the exclusion of 70 articles and the inclusion of 36 studies for the qualitative synthesis among which 4 studies allowed us to perform a meta-analysis.

Following the final selection of 36 articles, a data-charting form was jointly developed by the team and bibliographic details of the selected articles were registered in **Figure 1**. To classify the data, four categories were defined: (1) memory subsets, (2) senescence and exhaustion-associated markers, (3) cytokines, and (4) telomere length. Each section was assigned to teams of two or more reviewers that extracted the required information in the data-charting sheets. Where possible, the mean/median, standard deviation (SD)/standard error (SE), and the number of events recorded in flow cytometry were registered. If a value was missing, the authors were contacted to provide the details. In situations of lack of response from the authors, a qualitative description of the results was done.

Different domains of results were made: (i) for memory T cell subsets, Naïve (N), Central Memory (CM), Effector Memory (EM), and Terminal Effector (TE) subsets were defined by markers such as CD27, CD28, CCR7, CD45RA, CD45RO, and CD95; (ii) CD57, KLRG1, PD1, CTLA4, TIM3, LAG3, p16, p21, and γH2Ax were defined as immunosenescence and/or exhaustion markers; (iii) intracellular IFNγ, TNFα, IL-2, IL-4, IL-6, IL-10, granzyme B, and perforin were analyzed. Finally, (iv) telomere length was reported as Kb or MFI by flow cytometry. Some articles reported an additional group referred to as “super-old” which was also extracted when available.

### Risk of Bias Assessment

**Figure 2:**
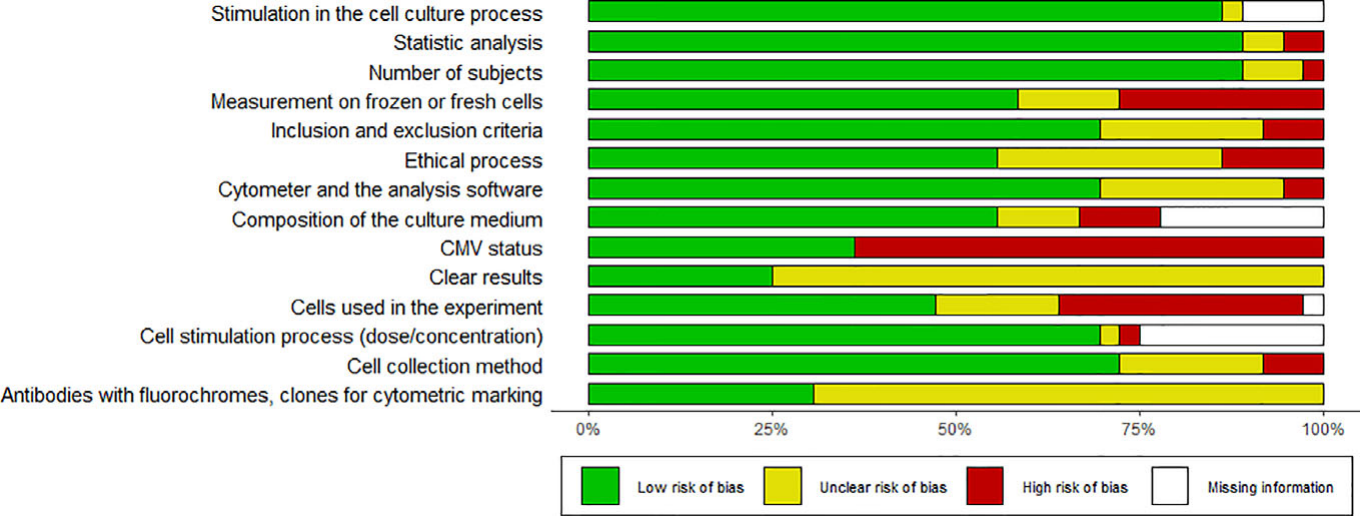
Risk of bias summary. Shows the bias results of all papers reviewed.

**Figure 3:**
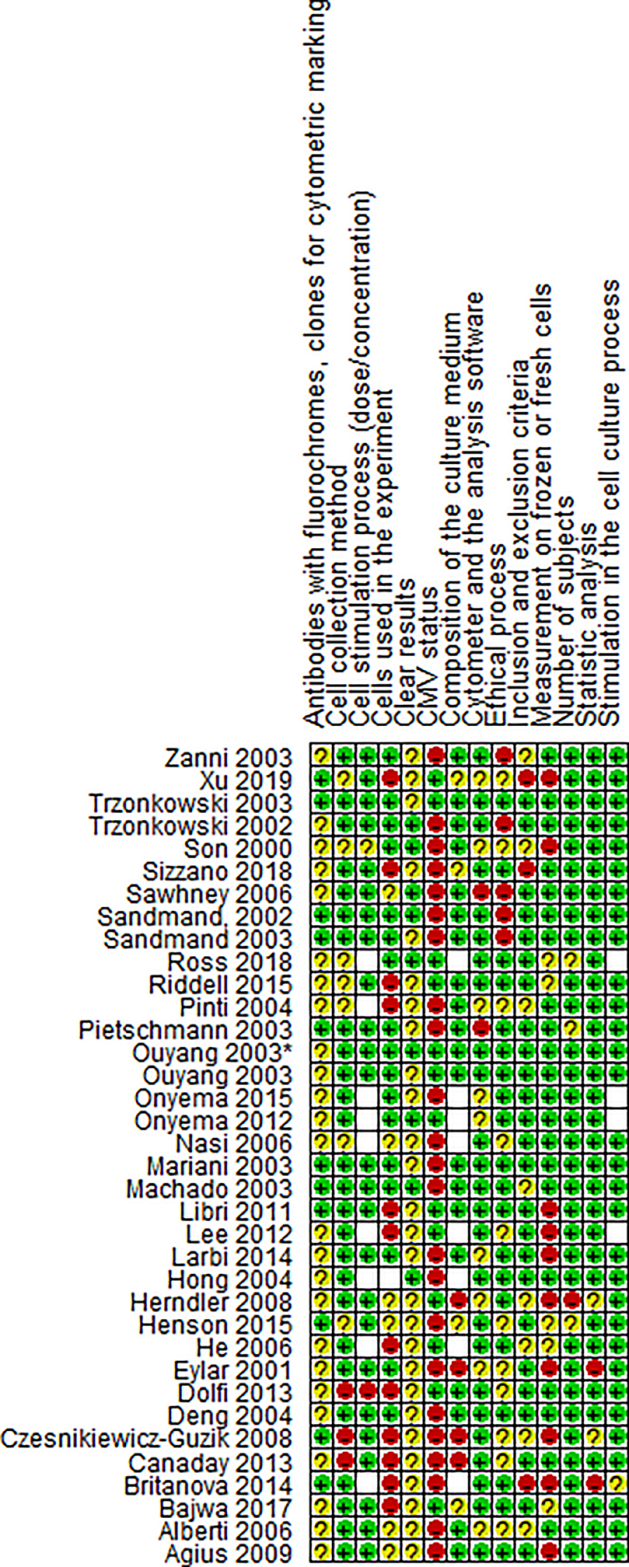
Risk of bias summary for paper. Review authors’s judgments about each risk of bias item for each included study.

We did not find a standard assessment tool to evaluate the risk of bias and quality of basic research articles in immunology. Consequently, we designed a table with 14 questions to detect the risk of bias associated with selection, design, methodology, and results (see **Table S2**). For this review, we considered that the articles assessing CMV status without bias were the ones that performed the screening and defined the positive and negative groups. Two reviewers independently scored articles according to description quality as: completely (1), partially (0.5) or not reported (0) and 0 to 100 percent rating was given, with 100% rating for the lowest risk of bias. The analysis was carried out globally and presented in **Table S2**. The risk of bias is illustrated in **Figures 2** and **3**.

### Synthesis of Results

Only four articles had the data required for a meta-analysis. This information was synthesized in forest plots. A qualitative description of the remaining papers was done and presented in a narrative form using tables and figures. Where possible, funnel plots were used to assess potential small study bias in meta-analysis containing ten or more studies and absence of statistical heterogeneity (14). Nonetheless, this was not possible due to lack of papers suitable for meta-analysis in our study.

### Statistical Analysis

The data available for quantitative analysis was frequency of the memory T cell populations. We used the mean and SD of each specific group count to calculate the standardized mean (SM) and standardized mean difference between groups. The summary estimate of effect had 95% confidence interval for each comparison. Weightings were done by the inverse variance method. Effects were summarized by the random effects meta-analysis. Effect size and confidence intervals of global and subgroup analysis were plotted using forest plots. There was no sufficient data to conduct a sensitivity analysis.

We assessed heterogeneity by visual inspection of the forest plot and quantified it using the I^2^ statistic, which describes the proportion of variability in the meta-analysis explained by heterogeneity of the studies rather than sampling error. Values ≥50% indicate significant heterogeneity between studies in the meta-analysis.

## Results

### Study Selection and Characteristics

The search strategy performed in this article led to an initial selection of 444 potential articles. 321 articles were retrieved from PUBMED/MEDLINE, 123 from EBSCO, none from BVS in the identification phase. After duplicates were removed, a total of 276 studies were identified (**Figure 1**). Out of these studies, 166 articles were excluded based on title and abstract. Further assessment of the remaining 110 full text articles led to the exclusion of 74 articles (**Table S3**) and inclusion of 36 studies for the qualitative synthesis, out of which, 4 studies were eligible for the performance of meta-analysis (15–50).

Characteristics of all included studies are shown in **Table S4**. All studies were characterized by at least two groups of interest (young and old) and experiments done on T cells obtained from peripheral blood and that these cells were used immediately for analysis or frozen for later measurement. Of the 36 studies included, 17 measured CD4 and CD8 T cells; 15 measured CD8; three measured CD4; and one measured γ/δ T cells. 15 studies measured changes in memory subsets, 21 measured markers associated with senescence and exhaustion, 15 measured cytokine expression, and five measured telomere length. 28% of the studies considered CMV serology. In these articles, measurement of intracellular cytokines was performed *via in vitro* stimulation, while analysis of markers and memory subpopulations were performed *ex vivo*.

### Risk of Bias Within Studies

Heterogeneity in methodology is a risk of bias for evaluating evidence in this type of study. There was high variability among the included studies. For instance, cryopreservation can affect the expression of membrane proteins and intracellular molecules which are used for the classification of memory subsets and identification of senescence markers. Only 28% of the studies did not describe whether the measurement was done on frozen or fresh cells (15, 22, 23, 26, 28–31, 49, 50); 33% of the articles did not report the number of cells used in each experiment (15, 17, 18, 20, 22, 24–26, 28, 31, 35, 36), while only 31% clearly described the antibodies with fluorochrome and clone used (15, 21, 22, 28, 31, 39, 41, 43, 44, 46, 48). The factor with the highest risk of bias is the clarity in the presentation of the results, since only 25% of the studies reported measures of central tendency (16, 27, 33, 37, 41, 42, 47, 48, 50). Most of the studies reported only the graphics and p-value, thus making it difficult to quantitatively compare results and rate evidence. It would be useful to standardize study protocols in order to obtain reliable results that can be compared.

### Synthesized Findings

#### Memory T Cell Subsets

As shown in **Table 1**, among the 36 eligible studies, 15 characterized T cell subsets with different combinations of defining markers. Of these studies, eight (53.3%) reported memory T cells using CD27 and CD45RA (15, 18, 20–23, 25, 28); four (26.6%) used CCR7 and CD45RA (26, 31, 32, 37); two (13.3%) used CD28 and CD95 (34, 45); and one (6.6%) used CCR7 and CD45RO (17), thus confirming the lack of homogeneity for determination of memory subpopulations of T cells. Only five studies (33.3%) reported donor CMV status and memory subsets conjointly (15, 18, 20, 26, 28). For CD8 T cells, 12 studies reported memory subset changes. Six of the studies reported memory subsets exclusively in CD8 T cells (20, 21, 25, 26, 37, 45), six reported differences in CD4 and CD8 T cells (17, 18, 22, 23, 31, 32), two studies reported memory changes exclusively in CD4 T cells (28, 34), and one study exclusively reported changes in γ/δ T cells (15). Herein, we report the subset frequency trends across studies by comparing the young and old populations (**Table 1**).

**Table 1:** Characteristics of studies reporting memory subsets.

No.	References	Year	Lymphocyte definition	Elderly	Young	CMV status	Memory definition	Stimulation	Elderly (mean ± SD, %)				Young (mean ± SD, %)			
				n	n				N	CM	EM	E	N	CM	EM	E
1	Xu et al. (15)	2019	γ/δ	12	12	Yes	CD27 CD45RA	No	↓	NS	NS	↑	↑	NS	NS	↓
2	Sizzano et al. (17)	2018	CD4/CD8	7	7	No	CCR7 CD45RO	No	NS	NS	NS	NS	NS	NS	NS	NS
3	Bajwa et al. (18)	2017	CD4/CD8	103	48	Yes	CD27 CD45RA	No	NC	NC	NC	NC	NC	NC	NC	NC
4	Riddell et al. (20)	2015	CD8	125		Yes	CD27 CD45RA	No	↓	NS	NS	↑	↑	NS	NS	↓
5	Henson et al. (21)	2015	CD8	8	8	No	CD27 CD45RA	Yes	NC	NC	NC	NC	NC	NC	NC	NC
6	Britanova et al. (22)	2014	CD4/CD8	7	10	No	CD27 CD45RA	No	18,8 ± 12,6	NM	NM	NM	37,5 ± 8,1	NM	NM	NM
7	Larbi et al. (23)	2014	CD4/CD8	15	15	No	CD27 CD45RA	No	↓	NS	↑	↑	↑	NS	↓	↓
8	Dolfi et al. (25)	2013	CD8	5	5	No	CD27 CD45RA	No	↓	NC	NC	NC	↑	NC	NC	NC
9	Lee et al. (26)	2012	CD8	43	62	Yes	CCR7 CD45RA	No	NC	NC	NC	NC	NC	NC	NC	NC
10	Libri et al. (28)	2011	CD4	± 67	± 40	Yes	CD27 CD45RA	No	↓	↑	↑	↑	↑	↓	↓	↓
11	Czesnikiewicz-Guzik et al. (31)	2008	CD4/CD8	26	31	No	CCR7 CD45RA	No	↓	↓	↓	↑	↑	↑	↑	↓
12	Nasi et al. (32)	2006	CD4/CD8	10	12	No	CCR7 CD45RA	No	↓	↑	↑	NS	↑	↓	↓	NS
13	Alberti et al. (34)	2006	CD4	20	12	No	CD28 CD95	No	35,6+/- 2,3	45,9+/- 2,8	11,8+/- 1,3		55 +/- 3,6	34,8+/- 2,8	8+/- 2,4	
14	Hong et al. (37)	2004	CD8	17	17	No	CCR7 CD45RA	No	9.5 +/- 2.6	NS	51.3 +/- 3.4	32.3 +/- 3.7	46.4 +/- 4.1	NS	28.2 +/- 2.8	18.0 +/- 2.5
15	Zanni et al. (45)	2003	CD8	10	10	No	CD28 CD95	No	3.6 +/- 1.4	42.3 +/- 6	54 +/- 6		40.6 +/- 5	36.6 +/- 5	16.8 +/- 9	

1. Information on Vδ1, mean ± SD not reported, Y were CMV- and O were CMV+ (**Figure 1B**) 2. The significant difference in the percentages of CD4 and CD8 subsets was between young and semi supercentenarians (**Figure 1**) 3. The distributions of CMV-specific CD4 and CD8 T cells among the memory compartments (**Figure S3A**) 4. Results from multiple linear regression fitting age and CMV response as covariates for CD8+ T cell subset composition (1B/**Table S1**) 5. Senescent characteristics in the memory subsets (**Figure 1**) 6. Values in CD8 (**Table 1**) 7. Impact of aging on T cell phenotype and function, values in CD8 (2A and 2 B) 8. Senescent and inhibitory characteristics in the memory subsets (**Figure S1**) 9. Association of CMV infection with the frequency of CD8+ T cell subsets in young and elderly people. (**Figure S1**) 10. Frequencies of each population within total CD4+ T cells are represented by grouping via age and CMV status, changes in N, EM and E subsets were CMV dependent (**Figure 1C**) 11. Values in CD8 T cells (**Figures 3B, D**) 12. The old group is centenarian (**Figure 2**) 13. Stimuli with PMA/ionomycin did not change CD95 and CD28 expression. Stimulation was carried out to analyze modifications in the intracellular production of cytokines (**Table 1**) 14. (**Figure 1B**) 15. Stimuli with PMA/ionomycin did not change CD95 and CD28 expression. Stimulation was carried out to analyze modifications in the intracellular production of cytokines (**Table 1**). The figures and tables correspond to the original article cited. N, naive; CM, central memory; EM, effector memory; E, effector; ↑, increased; ↓, reduced; NS, no changes or no significant changes between the groups; NM, not measured; NC, not compared between the groups; NR, not reported.

##### CD8 T Cells

12 studies explored CD8 T cell subsets (100%). Regarding the naïve compartment, eight (66.6%) reported a higher frequency of naïve CD8 T cells in young adults compared to older adults (20, 22, 23, 25, 31, 32, 37, 45), and just Riddel et al., evaluated CMV status, and both CMV+ and CMV- groups showed this higher frequency in young adults (20). Among the other four studies (33.3%), three did not compare naïve subsets between young and older adults (18, 21, 26), whereas one (8.3%) showed non-significant changes between the groups, and this particular study did not take CMV status into consideration, which could be altering the conclusion drawn by the researchers (17).

**Figure 4:**
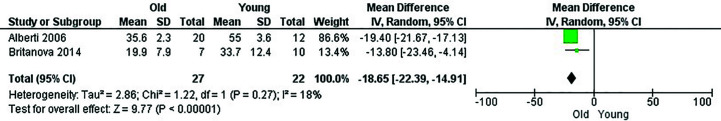
Influence of age on naive CD8+ T cell frequency. Forest plot for the different outcomes regarding cell frequency between old and young groups. The forest plot displays the SMD (squares) and 95% confidence interval of the individual studies. The diamond in each plot indicates the overall estimate and 95% confidence interval.

In this item, only three papers (22, 37, 45) were eligible for the performance of meta-analysis, which revealed that CD8 naïve T cells had a higher frequency in young adults compared to older adults, with a mean difference of 33.86 (95% CI = 28.40–39.32, *p* < 0.00001). However, none of these reported CMV status, so the decrease in naive cells in older adults could also be due to CMV infection and not only age. Heterogeneity was high (I^2^ = 82%). Effect analysis showed a direction toward the older adults and significant effect of age. Super-olds were not addressed for CD8 T cells due to lack of information (**Figure 4**). Of note, Britanova et al. (22) did neither reported the cryopreservation process nor statistical analysis conducted. Neither Hong et al. (37, 45) nor Zanni et al. (37, 45) fully reported the antibodies along with their clone and fluorochrome used. In this regard, given the aforementioned risks of bias, results should be interpreted with caution.

The memory compartment is divided into CM and EM. CM CD8 T cells were especially heterogeneous between studies. Of these studies, three (25%) reported no significant differences between groups (17, 20, 37): once again, just Riddel et al., considered CMV status and found that even though there was no significant difference between CMV- young and older adults, in the CMV+ older adults there was a lower frequency of this population compared to their younger counterparts (20). This same finding was reported by another article (8.3%), however in this study, CMV status was not assessed (31).Two other articles (16.6%) showed increased CM CD8 T cells in older adults compared to young adults (32, 45). The remaining six (50%) articles did not compare between groups (41.6%) (18, 21, 25, 26) and neither showed significant changes (8.3%) (23) nor interrogated this subpopulation (8.3%) (22). As stated for the naive compartment, these results are highly biased due to a lack of CMV status consideration given that only one of the seven articles that compared CM CD8 T cells, just one made the important distinction between CMV+ and CMV- adults.

On the other hand, EM CD8 T cells in three articles (26%) were elevated in older adults (23, 32, 37). In line with this, although Zanni et al. (8.3%) characterized T cells with CD28 and CD95, the bulk effector compartment was interrogated and it was found that effector CD8 T cells were also higher in the older adults (45). In contrast, Czesnikiewicz-Guzik et al. (8.3%) reported an increased EM cells in young adults (31). The remaining seven studies did not show any trend, since four (33.3%) of them did not compare between groups (18, 21, 25, 26); one (8.3%) did not show significant changes (17), and one (8.3%) did not interrogate this subpopulation (22). Interestingly, when the study included CMV status as a variable (8.3%), the CMV- older adults, unlike their CMV+ counterparts, had a statistically significant increase of this subset compared to CMV- young adults (20).

In four (33.3%) studies, TE CD8 T cells were increased in older adults (20, 23, 31, 37). This was observed in both CMV- and CMV+ groups, when this variable was included (20). Similarly, Zanni et al. (8.3%) reported a higher frequency of effector T cells in older adults (45). The remaining seven studies did not show any trend, since four (33.3%) of them did not compare between groups (18, 21, 25, 26); two (16.6%) showed no differences between groups (17, 32), and one (8.3%) did not investigate this subset (22) (**Table 1**).

##### CD4 T Cells

Among the eight articles reporting CD4 populations (100%), particularly for naïve T cell compartment, six (75%) studies reported significantly increased naïve subpopulation in young adults (22, 23, 28, 31, 32, 34). and just Libri et al., reported CMV status, showing that this increase was significant only for CMV+ donors (28). One (12.5%) of them reported non-significant changes (17), however as was mentioned for CD8 T cells, this lack of change could be explained by the low number of subjects and neglecting the CMV status of the participants, while the other (12.5%) did not compare between groups (18).

**Figure 5:**
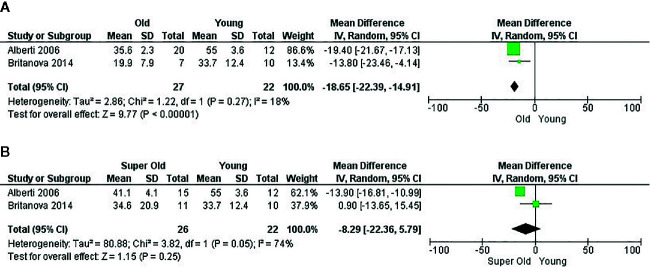
Influence of age on naive CD4+ T cell frequency. Forest plot for the different outcomes regarding cell frequency. **(A)** Changes in the frequency of CD4+ naive T cells between old and young groups and **(B)** Changes in the frequency of CD4+ naive T cells between super old and young groups.

Analysis of two papers (22, 34) which were eligible for performance of meta-analysis revealed that CD4 naïve T cells also had a higher frequency in young adults compared to older adults, with a mean difference of 18.65 (95% CI = 14.91–22.39, *p* < 0.00001). Unfortunately, neither of these articles measured CMV status of the participants, which leads to a significant bias of the mentioned effect. Heterogeneity was low (I^2^ = 18%). Effect analysis showed a direction toward the older adults and significant effect of age. When super-olds were addressed, heterogeneity increased (I^2^ = 74%) and the effect was not significant, presumably because of the SD in Britanova et al. (22) (**Figure 5**). Of note, Alberti et al. (34) neither reported the antibodies along with their clone and fluorochrome nor the cytometer and software analysis. In this regard, these results should be interpreted with caution.

As with CD8 T cells, the memory compartment is divided into CM and EM. In four (50%) articles, CM CD4 T cells showed an increased proportion in the aged group (23, 28, 32, 34), whereas this subset showed no differences between groups in two (25%) articles (17, 31). The remaining two (25%) studies neither compared between groups (18) nor interrogated this subpopulation (22). Interestingly, Libri et al. reported increased CM subset only in old CMV- donors (28).

Considering EM CD4 T cells, three (37.5%) articles reported higher frequencies in the older group (23, 28, 32). As in Zanni et al. in CD8 T cells, Alberti et al. (12.5%) reported the bulk effector compartment with CD28 and CD95 in CD4 T cells and found an increase in old adult (34, 45). The remaining four studies (50%) did not show any trend, since two (25%) of them showed non-significant changes (17, 31); one (12.5%) neither compared between groups (18) nor investigated this subset (12.5%) (22).

**Figure 6:**
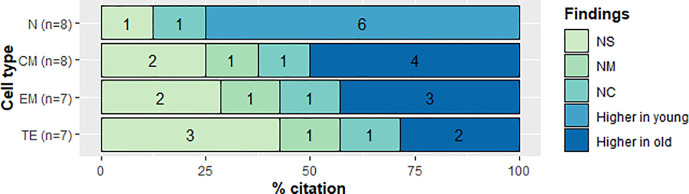
Qualitative analysis of frequency in CD4+ T cell subsets. CD4+ T cell subset and citation percent across studies comparing young and old populations. N, naive; CM, Central Memory; EM, Effector Memory; TE, Terminally Effector; NS, No Significant change; NM, Non measured; NC, not compared.

TE CD4 T cells were increased in older adults in two (25%) articles (23, 28). Similarly, Alberti et al. (12.5%) reported a higher frequency of effector T cells in older adults (34). The remaining five studies (62.5%) did not allow us to establish any trend, since three (37.5%) of them showed non-significant changes (17, 31, 32); one (12.5%) neither compared between groups (18) nor interrogated this subset (12.5%) (22) (**Figure 6**).

Of note, Libri et al. reported increased frequencies of EM and TE T cells in CMV-infected donors in both age groups as well as in old CMV+ compared to young CMV+ donors (28).

##### γδ T Cells

Among γδ T cells, those from young CMV+ adults had similar percentages of memory subsets with those of old CMV+ adults. In all three subsets of γδ T cells CMV- young adults had a higher frequency of naive cells than CMV+ adults. Concomitantly, an increase of TE cells was observed in the CMV+ young adult group (15) (**Table 1**).

#### Immunosenescence and Exhaustion Markers

21 out of 36 articles evaluated markers associated with immunosenescence and exhaustion. Of these studies, nine (42.8%) measured both CD4 and CD8 T cells (16, 24, 31–33, 36, 40, 41, 48), nine (42.8%) measured CD8 T cells (19, 21, 25, 27, 30, 35, 42, 44, 47), two (9.5%) measured CD4 T cells (28, 29), and one (4.7%) measured γδ T cells (15). Expression of these markers in different subsets of CD4 or CD8 T cells was determined in eight studies (38%) (15, 19, 21, 25, 28, 30, 33, 35, 36); 11 (52.3%) studies measured the total population of T cells (CD4 or CD8) (16, 24, 29, 31, 32, 40–42, 44, 47, 48), and the remaining two (9.5%) measured the total population and subsets of T cells (25, 27). Eight (38%) considered serology for CMV (15, 16, 27, 28, 35, 40, 42, 44) and six (28.5%) performed *in vitro* stimulation to measure expression of the markers (21, 24, 33, 40, 42, 44) (**Table 2**).

**Table 2:** Characteristics of studies reporting immunosenescence markers.

No.	References	Year	Lymphocyte definition	Elderly	Young	CMV status	Populations	Stimulation	Markers in elderly									
				n	n				CD28	CD57	KLRG1	CD95	PD1	CTLA4	TIM3	p16	p21	Others
1	Xu et al. (15)	2019	γ/δ	12	12	Yes	Subsets	No	NM	↑	↑	NM	NM	NM	NM	NM	NM	↑CD85j, ↑CD244+, NC : γH2AX
2	Ross et al. (16)	2018	CD4/CD8	10	9	Yes	Total	No	↓	NM	NM	NM	NM	NM	NM	NM	NM	↓CD31
3	Onyema et al. (19)	2015	CD8	11	11	No	Subsets	No	↓	↑	NM	NC	NC	NM	NM	NC	↑	↑CD245, NC Bcl-2
4	Henson et al. (21)	2015	CD8	8	8	No	Subsets	Yes	NM	↑	↑	NM	↑	NM	NM	NM	NM	↑ p38, ↑γH2AX
5	Canaday et al.	2013	CD4/CD8	24	24	No	Total	Yes	NM	NM	NM	NM	↓	↑	↓	NM	NM	↓ ICOS in CD4 *ex vivo*
6	Dolfi et al. (25)	2013	CD8	± 38	± 37	No	Total/subsets	No	NM	↑	↑	NM	↑	NM	NM	NM	NM	↑LAG-3 ↑2B4, ↑T-bet, ↑Eomes
7	Onyema O et al. (27)	2012	CD8	11	11	Yes	Total/subsets	No	↓	↑	↑	NM	NM	NM	NM	↑	↑	
8	Libri et al. (28)	2011	CD4	25		Yes	Subsets	No	NC	NC	NM	NM	NM	NM	NM	NM	NM	IL-7Rα, Bcl-2
9	Agius et al. (29)	2009	CD4	± 9	± 11	No	Total	No	NM	NM	NM	NM	NM	NM	NM	NM	NM	NS: CLA, CD11a, CCR4
10	Herndler-Brandstetter et al. (30)	2008	CD8	NR	NR	1.1.1 Yes	Subsets	No	NM	NS	NM	NM	NM	NM	NM	NM	NM	NS : CD62L, CD11a, CD126,
11	Czesnikiewicz-Guzik et al. (31)	2008	CD4/CD8	41	68	No	Total	No	↓	↑	NM	NM	NM	NM	NM	NM	NM	↑ CD85j, ↓ CD26
12	Nasi et al. (32)	2006	CD4/CD8	7	7	No	Total	No	NM	NM	NM	NM	NM	NM	NM	NM	NM	NS : CD127
13	Sawhney et al. (33)	2006	CD4/CD8	25	20	No	Subsets	Yes	NM	NM	NM	↑	NM	NM	NM	NM	NM	CD4: ↑CD95L; CD8: NS CD95L
14	He et al. (35)	2006	CD8	15	22	Yes	Subsets	No	↓	↑	NM	NM	NM	NM	NM	NM	NM	
15	Pinti et al. (36)	2004	CD4/CD8	14	13	No	Subsets	No	NM	NM	NM	↑	NM	NM	NM	NM	NM	
16	Ouyang et al. (40)	2003	CD4/CD8	5	5	Yes	Total	Yes	↓	NM	NM	NM	NM	NM	NM	NM	NM	NS: HLA-DR, CD69, CD45RO/RA
17	Machado et al. (41)	2003	CD4/CD8	± 10	± 20	No	Total	No	NM	NM	NM	NM	NM	NM	NM	NM	NM	↓ P-glycoprotein 1
18	Ouyang et al. (42)	2003	CD8	70	11	Yes	Total	Yes	NM	NM	↑	NM	NM	NM	NM	NM	NM	
19	Trzonkowski et al. (44)	2003	CD8	91	63	Yes	Total	Yes	↓	↑	NM	NM	NM	NM	NM	NM	NM	
20	Trzonkowski et al. (47)	2002	CD8	65	31	No	Total	No	↓	NM	NM	NM	NM	↓	NM	NM	NM	
21	Sandmand et al. (48)	2002	CD4/CD8	15	26	No	Total	No	NM	NM	NM	↑	NM	NM	NM	NM	NM	

1. Information of Vδ1mean ± SD not reported (**Figures 1I**, **3B**), 2. (**Figure 2A**). 3. Expression of the different markers among subpopulations of CD28/CD57, (**Figures 2**, **4**). 4. The T cell subsets from old individuals express a greater array of senescent markers relative to young individuals (**Figure 1**), 5. Dates shown in CD8 (**Figure 1D**), 6. Dates in Total CD8 T cell (**Figures 1A**, **2A**, **3B**), 7. Only in CD28^−^ CD57+ cells a significantly higher p16 expression was found in the elderly compared to the Young subjects (**Figure 2B**, 3 y 4), 8. (**Figure 3A**), 9. (**Figure S1**), 10. CD8+CD45RO+CD25+ from elderly vs CD8+CD45RA+CD28+ from young (**Figure 1B**), 11. Dates in total CD8 T cells (**Figures 2A**, **5A**), 12. (**Figure 5**. and text) 13. (**Figures 2**, **3**, **4**), 14. CD57 increased and CD28 decreased in tetramer negative CD8 T cells, NS in tetramer-positive cells (**Figures 2B, C**, **3B, C**), 15. No increase observed at single-cell level on CD45RA negative T lymphocytes (**Figure 4**), 16. CMV peptide-specific CD8 (**Table 1**), 17. (**Figures 1**, **2**), 18. (**Figure 3B**), 19. Comparison between young responders and old (**Figure 1A**), 20. (**Table 1**), 21. There was no difference between the two elderly groups (Centenarians >100 years, n=25), (**Table 1**). The figures and tables correspond to the original article cited. ↑, increased in older; ↓, reduced in older; NS, no changes or not significant changes between the groups; NM, not measured; NC, not compared between the groups; NR, not reported.

The main markers evaluated were CD57 (47.6%), CD28 (42.8%), KLRG1 (23.8%), CD95 (19%), PD-1 (19%), CTLA-4 (9.5%), p16 (9.5%), p21 (9.5%), γH2AX (9.5%), CD85j (9.5%), and CD11a (9.5%) (15, 16, 19, 21, 24, 25, 27, 28, 30, 31, 33, 35, 36, 40, 42, 44, 47, 48). The following molecules were evaluated in one article at a time: CD127, CD31, CD244, CD245, Bcl-2, p38, ICOS, LAG-3, TIM-3, 2B4, T-bet, Eomes, IL-7Rα, Bcl-2, CLA, CCR4, CD62L, CD126, CD26, CD95L, HLA-DR, CD69, and P-glycoprotein 1 (15, 16, 19, 21, 24, 25, 28–33, 40, 41).

In γδ T cells, the CD57 expression significantly increased in aged subjects CMV+ in Vδ1+ and Vδ1−Vδ2− subsets, but no significant differences in young CMV- and CMV+ in any subsets (15). CD8 T cells expressing CD57 were more frequent in older adults (19, 21, 25, 27, 31, 35, 44). The expression of this marker on various memory subsets was increased in TE population in both age categories. However, this happened to a lesser extent in young adults (21). In CD4 T cells, upregulation of CD57 was highly correlated with age (31). Likewise, the expression of CD57 was higher in TE and EM subsets than N and CM subsets in CMV seropositive subjects (28).

The percentage of CD8 and γδ T cells (Vδ1+ and Vδ1−Vδ2−) expressing KLRG1 was increased in older adults (15, 21, 25, 27, 42). Likewise, CD95 expression was increased in CD8 T cells from older adults compared to young adults (33, 36, 48). CD95 expression was highest in TE when compared to the other memory subsets in CD8 T cells (19). The frequency of CD95 expressing CD4 T cells was higher in older adults than in young adults (33, 36, 48).

On the other hand, CD28 expression decreased in CD8 T cells from older adults compared to young adults and was correlated with CMV+ status (16, 19, 27, 31, 35, 40, 44, 47). Loss of CD28 expression is more frequent in CD8 than CD4 T cells (31). Libri et al. reported low CD28 expression in EM and TE CD4 T cells compared to naïve and CM T cells (28). In general, CD4 T cells are more resistant to age-associated phenotypic changes than CD8 T cells (31).

Among the studies selected for this review, only four measured the expression of PD-1 (19, 21, 24, 25). Two reported upregulation of PD-1 in older adults compared to young adults (21, 25). In addition, PD-1 expression was upregulated in CM and EM subsets, showing that PD-1 is augmented in old and differentiated populations (19, 21, 24, 25). Interestingly, Canaday et al. reported lower expression of PD-1, higher expression of CTLA-4, and lower expression of Tim-3 in older adults (24). Dolfi et al. also reported an increased expression of LAG-3 in T cells from older adults compared to young adults (25). Due to heterogeneity among study design, these reports are not conclusive.

Proteins p16, p21, and γH2AX were measured in few articles (19, 21, 27); however, all articles reported an increased expression of these molecules in older adults. Considering the lack of studies on these molecules, it was not possible to assume their utility for identifying senescent T cells, even when these molecules have been considered as distinctive of replicative senescence in other cell types.

#### Cytokines

Of the 36 eligible studies, 15 reported measurement of different cytokines and serine proteases such as IFNγ, TNFα, IL-2, IL-4, IL-6, IL-10, granzyme, and perforin. Of these studies, 12 measured at least one of the above mentioned cytokines in CD8 T cells; seven in CD4 T cells, and one in γδ T cells, considering the total populations or subsets by memory, markers or antigen specificity (**Table 3**).

**Table 3:** Characteristics of studies reporting cytokine.

No.	References	Year	Lymphocyte definition	Elderly	Young	1.1.2 CMV status	Populations	Stimuli	Cytokines							
				n	n				IFNγ	TNFα	IL-2	IL-4	IL-10	Granzyme B	Perforin	Others
1	Xu et al. (15)	2019	γ/δ	12	12	1.1.3 Yes	Vδ1/N vs E	1.1.4 PMA+ionomycin	↑	↑	↓	NM	NM	NM	NM	↑MIP-1α
2	Riddell et al. (20)	2015	CD8	8	8	1.1.5 Yes	Total/CD27 CD45RA	1.1.6 Anti-CD3 beads+IL-2	NS	NS	↑ CD8	NM	NM	NM	NM	
				7	7		CMV specific CD8/CD27 CD45RA	Peptide+IL-2	NS	NS	NS	NM	NM	NM	NM	
3	Henson et al. (21)	2015	CD8	8	8	1.1.7 No	CD27 CD45RA	1.1.8 Anti-CD3 beads	↑	↑	NM	NM	NM	↓	↓	
4	Larbi et al. (23)	2014	CD8	15	15	1.1.9 No	CD8	1.1.10 PMA+ionomycin	↑	↑	NM	NM	NM	NM	NM	
5	Dolfi et al. (25)	2013	CD8	44	54	1.1.11 No	1.1.12 Total/virus specific CD8	1.1.13 Peptide or SEF	↑	↑	↑	NM	NM	↓	NM	↓MIP-1β
6	Agius et al. (29)	2009	CD4	± 8	± 8	1.1.14 No	Total	1.1.15 Peptide	NS	NM	NM	NM	NM	NM	NM	
7	Alberti et al. (34)	2006	CD4	20	12	1.1.16 No	CD95 CD28	1.1.17 PMA+ionomycin	↓	↓	NM	NS	NM	NM	NM	
8	Deng et al. (38)	2004	CD4/CD8	11	5	1.1.18 No	CD4/CD8	1.1.19 Peptide	NS	NM	NM	NM	NM	NM	NM	
9	Ouyang et al. (42)	2003	CD8	70	11	1.1.20 Yes	CD8+KLRG1+ CD8+KLRG1−	1.1.21 PMA+ionomycin	↓KLRG1+	NM	NM	NM	NM	NM	NM	
10	Pietschmann et al. (43)	2003	CD4/CD8	79	75	1.1.22 No	CD4/CD8	1.1.23 PMA+ionomycin	↑CD8	NM	NM	↑	NM	NM	NM	
11	Trzonkowski et al. (44)	2003	CD8	91	63	1.1.24 Yes	CD8	1.1.25 Peptide then PMA+ionomycin	NM	NM	NM	NM	↑	NM	NM	
12	Zanni et al. (45)	2003	CD8	10	10	1.1.26 No	CD95 CD28	1.1.27 PMA+ionomycin	↑	↑	↑	↑	↑	NM	NM	↑ IL-6
13	Sandmand et al. (46)	2003	CD4/CD8	14	25	1.1.28 No	CD4/CD8	1.1.29 PMA+ionomycin	NM	↑	NM	NM	NM	NM	NM	
14	Sandmand et al. (48)	2002	CD4/CD8	14	24	No	CD4	PMA+ionomycin	↓	NM	NM	↑	NM	NM	NM	
							CD8		↑	NM	NM	↑	NM	NM	NM	
15	Eylar et al. (49)	2001	CD4/CD8	40	48	1.1.30 No	CD4/CD8	1.1.31 Antibody anti-CD3+PMA	↑ CD8	↑	NM	NM	NM	NM	NM	

1. Information of Vδ1mean ± SD not reported; NS in Vδ2+ ((**Figures 2A, D**), 2. Measurement of double and triple positive populations for IFN/Y, TNF-a and IL-2, finding an increase in the elderly in the percentage of total CD8+ producers of IL-2 and TNF-a (CD8+ IL-2+ TNF-a+), as well as an increase in CD8 + CMV + being these triple positive for IL-2, TNF-a and IFN-y (**Figures 5B, D**, **6B, D**), 3. ↑ IFN-y in CD27+CD45RA+, CD27−CD45RA− and CD27−CD45RA+; ↑ TNF-a in CD27+CD45RA+ and CD27− CD45RA+; ↓ Granzyme B in CD27+ CD45RA−, CD27− CD45RA− and CD27− CD45RA+; ↓Perforin in CD27+CD45RA+, CD27−CD45RA− and CD27−CD45RA+ (**Figure 3C**), 4. The capacity of CD4 T cells to produce cytokines no change between the groups. Data of a representative case (**Figure 2D**), 5. Stimuli with CMV showed similar results as in influenza stimuli. The ability to produce multiple cytokines simultaneously was decreased in the elderly compared with young (**Figures 5**, **S2B, S2C**). 6. Values in blood, IFN γ-producing CD4 in elderly in blisters is significantly reduced compared to young population (**Figure 2E**) 7. ↓IFN-y: CD95−CD28+ and CD95+ CD28+; ↓ TNF-a: CD95+ CD28+; Nonagenarians (n=15): ↓ IFN-y in CD4+ CD95−CD28+ and CD4+ CD95+ CD28+; ↑ IL-4 in CD4+ CD95+ CD28+ (**Figures 3**, **4**, **5**). 8. Lower mean fluorescent intensity of IFN-y in T cells compared with that in young subjects (**Figures 2B, D**). 9. (**Figure 4**), 10. ↑ IFNγ was just for old women; NS in IL-4- producing CD4 in women (**Figures 1**, **2**), 11. (**Figure 3**), 12. ↑IL-4: CD95+ CD28-; ↑IL-6: CD95+ CD28+ and CD95+ CD28-; ↑IL-10: CD95+ CD28− (**Figures 4**, **5**). 13. Centenarians (>100-year-olds, n = 21): Increase in TNF-a-producing cells among CD8+ cells, but not CD4+ cells (**Figure 2**), 14. Centenarians (>100-year-olds, n = 25): Increase in TNF-a and IL-4-producing cells among CD8+ cells and increased only in IL-4 for CD4 compared to young (**Figure 1**), 15. (**Table 1**). The figures and tables correspond to the original article cited. ↑ is increased in older. ↓, reduced in older; NS, no changes or no significant changes between the groups; NM, not measured; PMA, phorbol myristate acetate; SEF, Staphylococcus enterotoxin F.

For CD8 T cells, ten studies measured IFNγ (20, 21, 23, 25, 38, 42, 43, 45, 48, 49), seven measured TNFα (20, 21, 23, 25, 45, 46, 49), three measured IL-2 (20, 25, 45), three measured IL-4 (43, 45, 48), two measured IL-10 (44, 45), two measured granzyme B (21, 25), one measured perforin (21), and one measured IL-6 (45). In the case of IFNγ, 70% of the studies reported a higher percentage of IFNγ-producing CD8 T cells in older adults than young adults; 20% reported non-significant statistical differences (21, 23, 25, 43, 45, 48, 49), and 10% reported a decrease (42). In the latter, it should be noted that the measurement was performed in a subset which included the KLRG1 marker (42). For TNFα, IL-2, IL-4, IL-10, and IL-6, 85.7% (21, 23, 25, 45, 46, 49), 100% (20, 25, 45), 100% (43, 45, 48), 100% (44, 45), and 100% (45) of the studies found higher percentage of CD8 T cells producing these cytokine, respectively. Two studies evaluated CD8 T cells expressing two or more cytokines simultaneously (IL-2, TNFα, and IFNγ), thus finding a higher percentage of CD8+ IL-2+ TNFα+ (21) and CD8+ IFNγ+ TNFα+ (45) subpopulations in older adults. In addition, 100% of the granzyme B (21, 25) and perforin (21) studies showed a lower proportion of CD8 T cells producing these cytotoxic molecules. In the overall analysis, the percentages of cytokines producing CD8 T cells were higher, while the percentage of serine protease was lower in older adults.

Regarding CD4 T cells, there are major heterogeneous results. For IFNγ, four out of the six studies reported a non-significant difference between the age groups (29, 38, 43, 49), the remaining reported a lower level in older adults (34, 48). Three studies measured TNFα (34, 46, 49), with 66% of them (46, 49) showing an increase in these cytokines in the elderly, as opposed to one article (33%) that reported a decrease in older adults (34). Three studies measured IL-4 (34, 43, 46), with 66% (43, 46) showing an increase in older adults, while the remaining percentage reported non-significant differences between the groups (34).

In addition, four studies evaluated the relationship between cytokine production, memory phenotype, and age (20, 21, 34, 45). Henson et al. (21) reported that naïve and EM CD8 T cells from older adults appear more polyfunctional compared to those from young adults, while Riddell et al. (20) reported no significant difference between the groups. Moreover, Alberti et al. and Zanni et al. reported increases in both Th1 and Th2 cytokine-producing CD4 and CD8 in non-naïve T cells in older adults (34, 45).

Finally, only one study evaluated the aforementioned cytokines in γδ T cells, finding that older adults have a higher percentage of IFNγ and TNFα-producing γδ T cells and decreasing numbers of IL-2 producing cells (15).

#### Telomere Length

Five studies measured telomere length: three (60%) on CD8 T cells (20, 30, 39); one (20%) on CD4 and CD8 T cells (50), and one (20%) on γδ T cells (15). Three (60%) measured telomere length in total population of T cells (30, 39, 50), while the remaining two (40%) measured telomere length in memory T cell populations (15, 20) (**Table 4**). Xu et al. showed a decrease in telomere length in EM cells compared to naïve and CM cells; however, in Vδ1+ and Vδ1− Vδ2− subsets of γδ T cells, they also found that CD57+ cells had a significant shortening in telomere length in all γδ T cell types when compared to CD57−. No comparison was done between the age groups (15).

**Table 4:** Characteristics of studies reporting telomere length.

No	References	Year	Lymphocyte definition	Elderly	Young	1.1.32 CMV Status	Memory definition	Elderly (mean ± SD)				Young (mean ± SD, %)			
				n	n			N	CM	EM	E	N	CM	EM	E
1	Xu et al. (15)	2019	γ/δ	9		Yes	CD27 CD45RA/CD57+	NC	NC	NC	NC	NC	NC	NC	NC
2	Riddell et al. (20)	2015	CD8	27	38	Yes	CD27 CD45RA	5,7 ± 1,92	4,6 ± 1,42	4,0 ± 1,26	4,1 ± 1,32	10,1 ± 2,82	8,2 ± 2,78	7,0 ± 2,57	7,7 ± 2,73
3	Herndler-Brandstetter et al. (30)	2008	CD8	NR	NR	Yes	CD45RARO/CD28 CD25	5.1 ± 0.4				7.1 ± 0.1			
4	Mariani et al. (39)	2003	CD8	10	18	No	Total	↓				↑			
5	Son et al. (50)	2000	CD4/CD8	30	22	No	Total	CD4+: 6.7 ± 2 CD8+:5.5 ± 1.7				CD4+: 8.7 ± 1.7 CD8+: 6.9 ± 1.8			

1. Vδ1 decrease of telomere length from Naïve to EM (**Figure 3H**), 2. (**Figure 3B**), 3. CD45RO+CD25− from elderly vs CD45RA+CD28+ from young (**Figure 3**), 4. Young vs 80 years old (**Figure 3B**), 5. Third group: 80–94 years (n = 19) CD4: 6.2 ± 1.9; CD8: 5.1 ± 1.9 (**Table 1**). The figures and tables correspond to the original article cited. NS, no changes or no significant changes between the groups; NM, not measured; NC, not compared between the groups.

The studies that measured telomere length in CD8 T cells (20, 30, 39, 50) showed that the different memory subsets and whole CD8 T cells had significant reduction of telomere length in older adults compared to young adults. Riddell et al. stratified young and older adults based on their CMV status and found that young CMV+ adults had shorter telomeres in all memory subsets compared to their CMV- counterparts. In older adults, the difference between CMV+ and CMV- was not significant. In older adults, the difference between CMV+ and CMV- was not significant (20). Son et al. showed a reduction of telomere length in CD4 T cells as a function of increasing age (50). CD4 T cells from both young and older adults showed longer telomeres than CD8 T cells (50). Telomere shortening may be a useful marker for immunosenescence in T cells, but other mechanisms are also involved in restricting their proliferative capacity (20).

## Discussion

The generation and maintenance of antigen-specific memory T cells is crucial for long-term immune protection and effective vaccination. Four memory subsets have been canonically described: naïve, central memory (CM), effector memory (EM), and terminally-differentiated effector cells (TE) which can be characterized by presence of surface markers including CCR7, CD45RA/RO, CD27, CD28, CD62L, and CD95, among others (51, 52). Despite the fact that total numbers of T cells remain relatively constant with aging, significant changes have been observed in the composition of memory subpopulations (i.e., naïve vs. memory cells) (53).

All included studies that compared the distribution of memory populations between young and older adults found a considerable reduction of the naïve subset in older adults, both in CD4 (17, 22, 23, 28, 31, 32, 34) as seen in CD8 (17, 22, 23, 25, 31, 32, 37, 45), although the magnitude of the latter was much greater (31, 32). This finding is consistent with literature reviews in the field and the low percentage of naïve cells could partially explain the increased susceptibility in older adults to new infections and development of malignant pathologies (54, 55). As shown in **Figures 4** and **5**, a meta-analysis was performed with four studies and significant results were only found when comparing the naïve subset from young and older adults. However, the risk of bias was high in two of these studies (**Figure 3**); therefore, these results should be evaluated with caution.

One of the factors that most contributed to this heterogeneity is the definition of age groups. Populations designated as “young adults” were aged 18 to 44 years and populations of “older adults” were aged 60 to 107 years. Similarly, the creation of subgroups within the older adults to assess whether the observed differences increased linearly with age is also done arbitrarily and adds to this high heterogeneity, thus negatively affecting integrative analysis in the field of immunosenescence (**Table 5**). The highest variability was observed in the “older” group, whose cutoff point was 70 (22, 32), 80 (17–19, 21, 23, 34), 90 years (28, 31, 45) or adults ≥65 years of age (15, 20, 25, 26, 37). In the “super-olds” classification, adults aged 70 to 100 years were included (18, 22, 34) and the least variable classification was that of “centenarians”, which included adults aged 98 to 107 years (17, 32).

**Table 5:** Summary: suggestions for diminishing heterogeneity in the study of immunosenescence.

To Perform screening of CMV status since it is an associated confounder with aging. To Establish Consensus among experts to define age groups to be able to compare studies. To Be more descriptive in the research protocol detailing the stimuli, the number of cells, culture media, antibodies, fresh or frozen cells. To Report measurements and statistical values for all experiments (not just the p-value). To Show and report both positive and negative results.

The inclusion of age groups with ranges not greater than 10 years in research protocols would greatly facilitate the comparison between studies carried out by different groups within the field, thus making findings more significant. Another alternative that could improve the quality of immunosenescence research is the addition of longitudinal studies that correlate the percentages of memory populations with aging. Using this method, studies where non-statistical significance was found in comparisons between groups could find negative correlations between the percentage of CD4 and CD8 naïve cells with age (17, 20, 28), as well as positive correlations between CD4 memory cells (17, 28) and CD8 TE (17, 20) with age. In this way, a first approach to evaluate the overall effect of aging on different cellular subpopulations could be done.

However, these studies also have their limitations and should only be complementary to subgroup comparisons, since centennial populations may behave like young or middle-aged adults and differ from older adults. For instance, Britanova et al. (22) showed a decrease in naïve cells with aging in the correlation analysis, but long-lived adults had a higher percentage of naïve T cells than older adults, thus suggesting the existence of homeostatic mechanisms or healthy aging in long-lived adults.

Achieving a reduction in variability could aid a better understanding of the redistribution of memory populations since, unlike the naïve subpopulation, their behavior showed contradictory results in the studies included, which made it impossible to assess the global effect of aging on these populations. One of the factors that could affect the modulation of these populations is the presence of chronic infections such as CMV (56). This particular viral infection is known to impact and shape immune cell differentiation and exhaustion, acting in many cases as a confounder of age itself in immunosenescence studies (15). As stated before, only a third of all included articles in this systematic review reported donor CMV status as a screening method. Here, we sought to discuss the main results in light of their CMV status.

Multiple linear regression analysis performed by Libri et al. allow them to discriminate the effect of CMV seropositivity and age itself on CD4 T cell memory subset frequency and found that age and CMV serostatus both contribute to the decrease in naive T cells during ageing but the increase in CM, EM and TE T cells in old individuals is apparently the result of age itself for CM subset and primarily result of CMV infection for EM and TE subsets (28).

Riddel et al., using linear regression as well, showed a different distribution of memory subsets of CD8 T cells with age depending on the CMV status of the individuals. Although the reduction of the naive compartment and increase of the TE with age was a shared feature, the CM and EM subsets had different behaviors in the CMV+ and the CMV- groups, which could contribute to the understanding of the immunomodulatory effect of CMV infection (20). For instance, CMV+ older adults had not only a reduction of the naive compartment, but of the CM subset as well, suggesting that chronic infection has an effect on these two subsets, which are particularly important to respond to new infections and maintain long-lasting memory of vaccine-induced response, respectively. On the other hand, CMV- older adult cells seem to not differentiate all the way to TE cells, but some of them seem to be able to stay with an EM phenotype, since there was also an increase in older adults compared to young adults. This could provide CMV- older adults with a better suited response to reinfection, as well as diminishing the inflammatory environment produced by TE cells, indicating that CMV infection leads to “inflammaging” through TE induction (20, 26). Of note, Bajwa et al. only reported results in memory subsets following CMV-specific T cell expansion in seropositive individuals (18).

On the other hand, analysis of memory subsets within γδ T cells showed significant changes in the naive and TE compartments. Due to a lack of a seronegative CMV older adult group, an age-dependent change is not possible to infer. A decrease in the frequency of naive cells in all three subsets of γδ T cells between CMV+ and CMV- young adults shows the immunomodulatory impact of CMV infection on memory generation and maintenance. Concomitantly, an increase of TE cells was observed in CMV+ young adults, suggesting that CMV infection induces γδ T cells to differentiate. As for the CMV+ older adult, no significant changes were reported by Xu et al. in comparison to CMV+ young adults, showing that this change of proportions of the different memory subsets is dependent on CMV serostatus and not age (15).

Similarly, some authors (19, 21, 25) reported important functional changes in both naïve and memory populations, thus suggesting greater differentiation in cells from older adults. For this reason, the use of a third marker associated with differentiation and/or functionality such as CD57 or CD28 could complement memory assessment, thereby improving flow cytometry panels for study of immunosenescence and exhaustion (19, 27).

In literature, different markers have been reported to evaluate immunosenescence and exhaustion in T cells. These markers are expressed when cells start to activate and differentiate into effector populations and are therefore increased in people who have experienced a higher number of antigenic encounters throughout their life. One of these markers is CD28, a co-stimulatory molecule required for T cell activation that diminishes its expression under repeated antigen stimulation, making the cells less prone to correctly activate (19, 31). Furthermore, CD57 has been described as a marker of terminal differentiation, thus increasing in T cells from old people. Onyema et al. (19) used these two markers to classify CD8 T cells and found that, in young donors, 80% of the cells were CD28+CD57−, indicating a less differentiated and exhausted CD8 population. In old donors, this population was diminished, while an immunosenescent CD57+ phenotype was more prevalent.

Donors used in experiments carried out by He et al. (35) were all CMV+ and results observed as increased CD57 and decreased CD28 expressions in the older group were obtained exclusively in CD8 T cells A2-NLV tetramer negative, as opposed to reports from Ouyang et al. (40) that also showed decreased CD8 CD28+ T cells from seropositive old people but in tetramer-specific cells. Change of CD57 expression in γδ T cells was only statistically significant in older adults compared to CMV- young adults. Since there was no difference between CMV+ and CMV- young adults, or CMV+ young and older adults, age and CMV infection could have a synergic effect causing the upregulation of CD57 (15).

On the other hand, Pera et al. found a significant increase in CD28^−^ CD4 T cells in CMV^+^ individuals compared to CMV^-^ individuals. Similarly, the frequencies of CD28^−^ CD8 T cells were generally higher in CMV^+^ individuals. In contrast, the effect of age on CD28^−^ CD4 and CD8 T cells is small. They concluded that the diminished expression of CD28 in T cells strongly suggests that these observations reflect CMV-associated immunomodulation rather than normal immunosenescence (57).

KLRG1 is also expressed in differentiated cells and mediates inhibitory effects (7). The expression of this molecule was found to increase in T cells from older adults (15, 21, 25, 27, 42), suggesting that this marker could possibly reflect an immunosenescent state. CD95 is a multifunctional receptor that is upregulated in response to activation and, depending on signals from the environment, can induce cell death (33). An increase in this receptor was found in CD8 T cells from older adults compared to young adults (33, 36, 48). On these cells, expression of CD95 could regulate apoptotic signaling, thereby making senescent cells resistant to apoptosis. However, it is important to clarify that due to the heterogeneity of the studies, a definitive conclusion cannot be made.

Of note, It seems that Ouyang et al. (42) divided their study in CMV donor status known and CMV donor status not known. Results shown regarding increased KLRG1 in older people and reduced IFN-y in CD8 KLRG1+ T cells in our analysis were taken from the former because experiments carried out in the CMV donor status known group included increased KLRG1 expression in CD8 CMV-specific T cells in the old group.

The proteins PD-1, CTLA-4, and LAG-3 are inhibitory receptors that can be expressed after persistent antigenic stimuli, leading to functional exhaustion of T cells characterized by proliferative failure and loss of cytokine production (24). The p16 and p21 proteins belong to the cyclin-dependent kinases family which regulates cell cycle progression. Their expression can increase as a consequence of genotoxic stress and inhibiting cell proliferation as a preventive measure for malignant transformation (58). Similarly, γH2AX protein is activated in response to DNA damage. Although few studies were found to measure these molecules, they report an increased expression of these proteins in older group, in accordance to what is reported in literature as key characteristics of cellular senescence (21).

It should be noted that although some articles did report CMV status, their results did not fit our study design, for example, Onyema et al. reported differences in each age group when CMV status was considered for CD57, CD28, P16 and p21 expression, but these conclusions were drawn on CD3 gate only, before the subsequent gating process (27). Libri et al, reported low CCR7, CD28 and IL-7Ra and high CD57 expression on naturally CMV-expanded CD4 EM and TE T cells when compared to N and CM CD4 T cells (28).

These markers are surface or intracellular proteins whose expression may be affected due to methodological factors such as cryopreservation of cells and/or *in vitro* stimulation (59). Therefore, it is advisable to standardize the methodological designs, since they constitute a factor with a high risk of bias within our review, making it impossible to draw conclusions.

Moreover, these markers seem to be accompanied by dysregulated cytotoxic ability and reduced telomere length. This decrease is among the hallmarks of aging (60). In the few studies that included this measurement, it was found that there was a decrease in telomere length in the TE subset compared to the other memory populations, as well as in the older adults relative to the young adults. This decrease in telomere length is a consequence of replicative senescence due to repeated antigen exposure in TE cells and due to the accumulation of DNA damage throughout life in older adults. Telomere shortening has been associated with an increased risk of mortality (20). Of particular note, conclusions drawn from Herndler-Brandstetter et al. on telomere length could reflect clear-cut age derived immune changes as experiments were conducted exclusively on seronegative individuals (30). Despite being a characteristic marker of cellular aging in T cells, not many studies perform this measurement when evaluating immunosenescence. This can be explained by the difficulty of the technique, high cost, and requirement of highly qualified personnel (20).

In addition, these functional markers have been associated with a high production of proinflammatory cytokines in older adults (61); however, the results of our review showed a tendency toward an increase in the percentage of both pro- (IFNγ, TNFα, IL-2, IL-6) and anti-inflammatory (IL-4 and IL-10) cytokine-producing T cells and decrease in expression of serine proteases. In CD4 T cells, the percentages of TNFα and IL-4 producing cells in older adults were both high, thus, a predominance toward any of the helper profiles (Th1 or Th2) could not be determined. As for γδ T cells, only one study evaluated this population, in which a proinflammatory cytokine production increase was observed in older adults in the Vδ1 subpopulation. However, since it is a single study, it does not provide sufficient evidence for changes in cytokine production in these cells with aging from which conclusions can be made.

Even though the population included in this review were people reported as healthy and of a certain age range, other variables that present a valid comparison to identify whether intracellular cytokine measurement by flow cytometry can be used as marker for immunosenescence can be observed. Among these variables are the cytokines, culture stimuli, cell population, and techniques employed.

In literature, there is a list of both anti- and proinflammatory cytokines that have been associated with aging. In the case of proinflammatory cytokines, measurement is commonly done in CD8 T cells and the main cytokines measured are IL-6, TNFα, IL-2, IL-1B, IL-8, IL-18, and IFNγ (62). However, the studies reviewed in this study only account for four out of seven cytokines, with IFNγ cytokine being the most frequently measured, leaving aside cytokines such as IL-6, which has been classically reported to be increased in serum of old individuals (63). On the other hand, in the case of anti-inflammatory cytokines, IL1-Rα, IL-4, IL-10, and TGFβ are reportedly measured in both CD4 and CD8 T cells (62); nevertheless, we found that only five studies measured IL-4 or IL-10, while the remaining studies measured none. Finally, although the assessment of serine proteases is related with the cytotoxic profile of CD8 (64), only two studies measured perforin and granzyme B. Production of this cytolytic molecule seems to be modulated mainly by age, given that CMV+ older adults secreted lower levels of this cytokine than both CMV- and CMV+ young adults. However, a definitive conclusion cannot be drawn since there was no a CMV- older adult group (15). This shows that there is still no consensus on cytokines that should be measured for the assessment of aging.

The stimulus for induction of cytokine production is of great importance since it has been reported that variations in culture conditions, as well as the type and time of stimulation can influence the production of cytokines (65, 66). For instance, the three studies that evaluated polyfunctionality showed different results and, when looking at the culture conditions, it is done differently in each of them. In the case of Dolfi et al., specific stimulation with viral peptides was performed, while in the case of Riddell et al. and Henson et al., non-specific stimulation, anti-CD3 pearls and IL-2, and PMA/ionomycin were respectively performed (20, 21, 25).

Another important element for getting comparable and reproducible results is the cell population in which the measurement is made. Two options were mainly found in this review: measurement in total populations of T cells (CD4/CD8/γδ) and specific antigen populations. As an example, Riddell et al. and Dolfi et al. evaluated the response of CD8 T cells to a specific stimulus with CMV peptides (20, 25). In the case of Riddell et al., the response of specific CMV T cells was directly measured by tetramers, finding an increase in the population that co-produced IL-2/TNFα/IFNγ and IL-2/IFNγ to a greater extent in the older adults (20). On the other hand, Dolfi et al. reported non-significant differences between young and old individuals despite performing the same stimulus with CMV peptide. The latter could be explained by the measurement being made on total CD8 instead of antigen-specific CD8 T cells (25).

The technique for measurement of cytokines considered in this study was flow cytometry, which is performed by measuring intracellular cytokines, inhibiting vesicular transport or at the level of a cell culture supernatant using a cytometric bead array. However, cytokine measurement can be performed using other techniques such as ELISA or ELISPOT which can detect molecules in the plasma of an individual, supernatant of a culture, or directly in reactive cells. Evidence has shown that these techniques are complementary rather than comparable, since they do not have a direct correlation with their results (67). Therefore, there is no standardized method of cytokine measurement, such that studies based exclusively on one of these techniques do not represent the outlook of cytokines as a marker of immunosenescence.

Our systematic review has some limitations. Age groups across studies included a fair degree of variability. Similarly, 75% of the studies did not report values in the results of the experiments (i.e., number of cells used, mean or median, SD, among other descriptives) and, despite contacting the authors, we were unable to access majority of the data. Consequently, only one meta-analysis, in one of our result domains, namely, the memory T cell subsets, could be done throughout our analysis. This limitation suggests that the heterogeneity between the age ranges of the study groups and variation between the protocols does not allow robust conclusions.

To reduce the variability of the data and make accurate comparisons between the studies, only studies with flow cytometry analysis were taken into account, excluding several studies, especially those reporting cytokines or cytoplasmic proteins that are usually measured by other experimental techniques. The non-distinction between fresh and frozen cells when carrying out or reporting the experiments may also influence the presence, absence or quantity of certain membrane proteins, as well as cytokine production and telomerase activity. Finally, most of the studies in basic sciences report the experiments in which their alternative hypothesis is verified, and rarely negative data is reported. We consider that this can generate a publication bias and we observe this phenomenon even in the absence of a feasible funnel plot construction.

## Conclusion

In conclusion, it was impossible to perform statistical analysis between the age groups for immunosenescence markers, cytokines, and telomere length due to the fact that data obtained from the included studies had high heterogeneity attributable to the different protocols employed. Therefore, we could not determine the best immunosenescence markers. However, based on the articles reviewed, we propose some essential items in the study of immunosenescence (**Table 6**). We considered that achieving methodological consensus in immunosenescence research is an aspect that requires special attention (**Table 5**).

**Table 6:** Recommendations for the study of immunosenescence in T cells by flow cytometry.

To evaluate memory, use a combination of markers that favor identifying at least four basic memory subsets such as CD45RA or CD45R0 along with CCR7 or CD62 and a third one to access memory in-depth, such as CD95 or CD57. Evaluate cytokine production measure: (a) Pro-inflammatory cytokines: one or more of the following IFN-y, TNF-a, or IL-2. (b) Anti-inflammatory cytokines: one or more of the following: IL-10 or IL-4. We consider that the decrease in CD28 expression and the increase in the expression of CD57 and KLRG1 are the ones that best describe an immunosenescent state. Telomere length is the feature that best describes T cells senescence. Although p16, p21, and H2AX are hallmarks of aging, not many studies use these markers to evaluate immunosenescence.

## Data Availability Statement

The original contributions presented in the study are included in the article/**Supplementary Material**. Further inquiries can be directed to the corresponding author.

## Author Contributions

IR: Coordination, design, search, selection, review of papers, and writing of the manuscript. NR, ML, LM, MM, JO, OR, and EV: Design, search, selection, review of papers, and writing of the manuscript. EH: Design, review, and correction of the manuscript. CP: Coordination, design, writing, and review of the manuscript. All authors contributed to the article and approved the submitted version.

## Funding

This work was funded by MinCiencias (contract numbers: 110177758253 and 110184168973) and Universidad Nacional de Colombia (Hermes numbers: 44597, 44596, 41790, and 42207).

## Conflict of Interest

The authors declare that the research was conducted in the absence of any commercial or financial relationships that could be construed as a potential conflict of interest.
