# Supplementary material for: Immunosenescence Study of T Cells: A Systematic Review
Source: Front Immunol. 2021 Jan 15;11:604591. doi: 10.3389/fimmu.2020.604591 (PMC7843425; doi:10.3389/fimmu.2020.604591)
Supplement: Supplementary file 1 [file Table_1.docx]

**Table S1. Search Strategy**

| Database | Strategy |
| --- | --- |
| PubMed | (((((("Humans"[Mesh]) AND ("Immunosenescence"[Mesh] OR "Aging"[Mesh]))) AND ((((((("Biomarkers"[Mesh]) OR surface protein) OR surface marker) OR protein marker) OR senescence marker)) OR "Cytokines"[Mesh])) AND ((((((("Lymphocytes"[Mesh]) OR "T-Lymphocytes"[Mesh]) OR "B-Lymphocytes"[Mesh]) OR "Killer Cells, Natural"[Mesh]) OR "Macrophages"[Mesh]) OR "Monocytes"[Mesh]) OR "Dendritic Cells"[Mesh])) AND (((((("Real-Time Polymerase Chain Reaction"[Mesh]) OR "Blotting, Western"[Mesh]) OR "Flow Cytometry"[Mesh]) OR "beta-Galactosidase"[Mesh]) OR "Fluorescent Antibody Technique"[Mesh] OR "Telomere"[Mesh] OR "Telomere Shortening"[Mesh]) OR "Telomerase"[Mesh]))) NOT (((("Patients"[Mesh]) OR "Disease"[Mesh]) OR "Animals, Laboratory"[Mesh]) OR "Review" [Publication Type]) |
| MEDLINE/EBSCO | (((((("Humans") AND ("Immunosenescence" OR "Aging"))) AND ((((((("Biomarkers") OR surface protein) OR surface marker) OR protein marker) OR senescence marker)) OR "Cytokines")) AND ((((((("Lymphocytes") OR "T-Lymphocytes") OR "B-Lymphocytes") OR "Killer Cells, Natural") OR "Macrophages") OR "Monocytes") OR "Dendritic Cells")) AND (((((("Real-Time Polymerase Chain Reaction") OR "Blotting, Western") OR "Flow Cytometry") OR "beta-Galactosidase") OR "Fluorescent Antibody Technique" OR "Telomere" OR "Telomere Shortening") OR "Telomerase"))) NOT (((("Patients") OR "Disease") OR "Animals, Laboratory") OR "Review" [Publication Type]) |
| BVS | (((((("Humanos") AND ("Inmunosenescencia" OR "Envejecimiento"))) AND (((((((("Biomarcadores") OR proteína de superficie) OR marcador de superficie) OR marcador de proteína) OR marcador de senescencia)) OR "Citoquinas")) AND (((((((("Linfocitos") OR "Linfocitos T") OR "Linfocitos B") OR "Células Asesinas, Naturales") OR "Macrófagos") OR "Monocitos") OR "Células dendríticas")) AND ((((((("Reacción en cadena de la polimerasa en tiempo real") OR "Blotting, Western") OR "Citometría de flujo") O "beta-Galactosidasa") OR "Técnica de anticuerpos fluorescentes" OR "Telómero" OR "Acortamiento de telómero") OR "Telomerasa"))) NO ((((("Pacientes") OR "Enfermedad") O "Animales, Laboratorio") OR "Revisión" [Tipo de publicación]) |
