# Supplementary material for: Immunosenescence Study of T Cells: A Systematic Review
Source: Front Immunol. 2021 Jan 15;11:604591. doi: 10.3389/fimmu.2020.604591 (PMC7843425; doi:10.3389/fimmu.2020.604591)
Supplement: Supplementary file 2 [file Table_2.docx]

**Table S2. Reporting risk of bias scheme**

|  |  | Completely (1) | Partly (0.5) | No reported (0) | Risk Unknow (-) |
| --- | --- | --- | --- | --- | --- |
| 1 | Was the number of subjects per experiment reported? | Yes | Not clearly reported | No | Not apply |
| 2 | Were the inclusion and exclusion criteria reported? | Yes | Not clearly reported | No | Not apply |
| 3 | Did the ethical committee approve the study, and participants provide written informed consent? | Yes | Not clearly reported | No | Not apply |
| 4 | Were the PBMC and T cells separation process reported? | Yes | Not clearly reported | No | Not apply |
| 5 | Did the authors describe if the measurement was made on frozen or fresh cells? | Yes | Not clearly reported | No | Not apply |
| 6 | Did the authors describe if the cells were stimulated? | Yes | Not clearly reported | No | Not apply |
| 7 | Was the process of the T cell stimulation described? | Yes | Not clearly reported | No | Not apply |
| 8 | Was the number of PBMCs or T cells used per experiment reported? | Yes | Not clearly reported | No | Not apply |
| 9 | Was the composition of the culture medium described? | Yes | Not clearly reported | No | Not apply |
| 10 | Were the antibodies, fluorochrome, and clone used described? | Yes | Not clearly reported | No | Not apply |
| 11 | Were the flow cytometer and software used described? | Yes | Not clearly reported | No | Not apply |
| 12 | Was the statistical analysis used described? | Yes | Not clearly reported | No | Not apply |
| 13 | Were the results shown clearly? | Yes | Not clearly reported | No | Not apply |
| 14 | Was the CMV status evaluated? | Yes | Not clearly reported | No | Not apply |
