# Supplementary material for: Immunosenescence Study of T Cells: A Systematic Review
Source: Front Immunol. 2021 Jan 15;11:604591. doi: 10.3389/fimmu.2020.604591 (PMC7843425; doi:10.3389/fimmu.2020.604591)
Supplement: Supplementary file 3 [file Table_3.docx]

**Table S3. Excluded articles**

| Study | | Reason for exclusion |
| --- | --- | --- |
| de Armas | **2020** (1) | Does not have control group |
| Tedone | **2020** (2) | Protocol |
| Zapata | **2019** (3) | Different cellular population |
| Louati | **2019** (4) | Does not have the age range of study |
| Rahmatpanah | **2019** (5) | Different cellular population |
| Lopes | **2018** (6) | Different cellular population |
| Kallemeijn | **2018** (7) | Does not have the age range of study |
| Fali | **2018** (8) | Different cellular population |
| Kaszubowska | **2018** (9) | Different cellular population |
| Frasca | **2017** (10) | Different cellular population |
| Goldeck | **2016** (11) | Different cellular population |
| Wang | **2016** (12) | Different cellular population |
| Najarro | **2015** (13) | Does not have control group |
| Kannan | **2015** (14) | Different cellular population |
| Bigley | **2015** (15) | do not have the age range of study |
| Holcar | **2015** (16) | do not have the age range of study |
| Frasca | **2015** (17) | Different cellular population |
| Tarazona | **2015** (18) | Protocol |
| Frasca | **2015** (19) | Protocol |
| Jergović | **2014** (20) | Does not have the age range of study |
| Chou | **2014** (21) | Does not have the age range of study |
| Busse | **2014** (22) | Does not have control group |
| Compté | **2014** (23) | Does not have control group |
| Prakash | **2013** (24) | Different cellular population |
| Alvarez | **2012** (25) | Markers not available |
| Orsini | **2012** (26) | Different cellular population |
| Guerra-Laso | **2013** (27) | Different cellular population |
| Priyanka | **2013** (28) | Does not have the age range of study |
| Garbe | **2012** (29) | Does not have control group |
| Frasca | **2012** (30) | Does not have the age range of study |
| Longo | **2012** (31) | Does not have control group |
| Hearps | **2012** (32) | Different cellular population |
| Yalcin | **2012** (33) | Does not have the age range of study |
| Ferrando-Martínez | **2011** (34) | Different conditions of comparison |
| Goetzl | **2010** (35) | Different cellular population |
| Kaszubowska | **2011** (36) | Different cellular population |
| Ogden | **2011** (37) | Different cellular population |
| Vilas-Boas | **2011** (38) | Markers not available |
| Marttila | **2011** (39) | Does not specify the condition of patients |
| Kimura | **2010** (40) | Does not have control group |
| Horvathova | **2009** (41) | Different conditions of comparison |
| Kushner | **2010** (42) | Does not have the age range of study |
| Caraux | **2010** (43) | Different cellular population |
| Panda | **2010** (44) | Different cellular population |
| Colonna-Romano | **2009** (45) | Different cellular population |
| Hoffmann | **2009** (46) | Different cellular population |
| Kilpatrick | **2008** (47) | Different conditions of comparison |
| Kaszubowska | **2008** (48) | Different cellular population |
| Simone | **2008** (49) | Does not have control group |
| Ghia | **2007** (50) | Does not have control group |
| Agrawal | **2007** (51) | Different cellular population |
| Mazzatti | **2007** (52) | Genetic expression |
| Njemini | **2006** (53) | Does not have the age range of study |
| Walrand | **2006** (54) | Different cellular population |
| Ju | **2006** (55) | Does not have control group |
| Kovaiou | **2005** (56) | Does not have the age range of study |
| Poggioli | **2004** (57) | Different cellular population |
| Larbi | **2004** (58) | Different methods of extraction |
| Simar | **2004** (59) | Different cellular population |
| Son | **2003** (60) | Different cellular population |
| Breitbart | **2002** (61) | Does not have control group |
| Tsaknaridis | **2003** (62) | Does not have control group |
| Kimmig | **2002** (63) | Does not have control group |
| Njemini | **2002** (64) | Different cellular population |
| Poggioli | **2002** (65) | Different cellular population |
| McNerlan | **2002** (66) | Different cellular population |
| Olaussen | **2001** (67) | Does not have control group |
| Ginaldi | **2000** (68) | Does not have the age range of study |
| De Martinis M | **2000** (69) | Does not have the age range of study |
| De Martinis M | **2000** (70) | Does not have the age range of study |
| Myśliwska | **2000** (71) | Different cellular population |
| Yen | **2000** (72) | Different cellular population |
| Beinert T | **2000** (73) | Different cellular population |
| Pietschmann | **2000** (74) | Different cellular population |

**References**

1. de Armas LR, Pallikkuth S, Rinaldi S, Pahwa R, Pahwa S. Implications of Immune Checkpoint Expression During Aging in HIV-Infected People on Antiretroviral Therapy. AIDS Res Hum Retroviruses. 2019 Nov/Dec;35(11-12):1112-1122. doi: 10.1089/AID.2019.0135.
2. Tedone E, Huang E, O'Hara R, Batten K, Ludlow AT, Lai TP, Arosio B, Mari D, Wright WE, Shay JW. Telomere length and telomerase activity in T cells are biomarkers of high-performing centenarians. Aging Cell. 2019 Feb;18(1):e12859. doi: 10.1111/acel.12859.
3. Zapata HJ, Van Ness PH, Avey S, Siconolfi B, Allore HG, Tsang S, Wilson J, Barakat L, Mohanty S, Shaw AC. Impact of Aging and HIV Infection on the Function of the C-Type Lectin Receptor MINCLE in Monocytes. J Gerontol A Biol Sci Med Sci. 2019 May 16;74(6):794-801. doi: 10.1093/gerona/gly209.
4. Louati N, Rekik T, Menif H, Gargouri J. Blood lymphocyte T subsets reference values in blood donors by flow cytometry. Tunis Med. 2019 Feb;97(2):327-334. PMID: 31539091.
5. Rahmatpanah F, Agrawal S, Scarfone VM, Kapadia S, Mercola D, Agrawal A. Transcriptional Profiling of Age-Associated Gene Expression Changes in Human Circulatory CD1c+ Myeloid Dendritic Cell Subset. J Gerontol A Biol Sci Med Sci. 2019;74(1):9‑15. DOI: 10.1093/gerona/gly106
6. Lopes AB, Lopes LB, da Silveira Antunes RN, Fukasawa JT, de A Cavaretto D, Calamita Z. Effects of Immunosenescence on the Lower Expression of Surface Molecules in Neutrophils and Lymphocytes. Current aging science. Bentham Science Publishers; 2018;11(2):118‑25.
7. Kallemeijn MJ, Kavelaars FG, van der Klift MY, Wolvers-Tettero ILM, Valk PJM, van Dongen JJM, et al. Next-Generation Sequencing Analysis of the Human TCRγδ+ T-Cell Repertoire Reveals Shifts in Vγ- and Vδ-Usage in Memory Populations upon Aging. Front Immunol. 2018;9:448. DOI: 10.3389/fimmu.2018.00448
8. Fali T, Fabre-Mersseman V, Yamamoto T, Bayard C, Papagno L, Fastenackels S, et al. Elderly human hematopoietic progenitor cells express cellular senescence markers and are more susceptible to pyroptosis. JCI Insight. 3(13). DOI: 10.1172/jci.insight.95319
9. Kaszubowska L, Foerster J, Kwiatkowski P, Schetz D. NKT-like cells reveal higher than T lymphocytes expression of cellular protective proteins HSP70 and SOD2 and comparably increased expression of SIRT1 in the oldest seniors. Folia Histochem Cytobiol. 2018;56(4):231‑40. DOI: 10.5603/FHC.a2018.0025
10. Frasca D, Diaz A, Romero M, D’Eramo F, Blomberg BB. Aging effects on T-bet expression in human B cell subsets. Cell Immunol. 2017;321:68‑73. DOI: 10.1016/j.cellimm.2017.04.007
11. Goldeck D, Oettinger L, Janssen N, Demuth I, Steinhagen-Thiessen E, Pawelec G. Cytomegalovirus Infection Minimally Affects the Frequencies of B-Cell Phenotypes in Peripheral Blood of Younger and Older Adults. Gerontology. 2016;62(3):323‑9. DOI: 10.1159/000382076
12. Wang Q, Westra J, van der Geest KSM, Moser J, Bijzet J, Kuiper T, et al. Reduced levels of cytosolic DNA sensor AIM2 are associated with impaired cytokine responses in healthy elderly. Exp Gerontol. 2016;78:39‑46. DOI: 10.1016/j.exger.2016.02.016
13. Najarro K, Nguyen H, Chen G, Xu M, Alcorta S, Yao X, et al. Telomere Length as an Indicator of the Robustness of B- and T-Cell Response to Influenza in Older Adults. J Infect Dis. 2015;212(8):1261‑9. DOI: 10.1093/infdis/jiv202
14. Kannan S, Kurupati RK, Doyle SA, Freeman GJ, Schmader KE, Ertl HCJ. BTLA expression declines on B cells of the aged and is associated with low responsiveness to the trivalent influenza vaccine. Oncotarget. 2015;6(23):19445‑55. DOI: 10.18632/oncotarget.4597
15. Bigley AB, Spielmann G, Agha N, Simpson RJ. The Effects of Age and Latent Cytomegalovirus Infection on NK-Cell Phenotype and Exercise Responsiveness in Man. Oxid Med Cell Longev. 2015;2015:979645. DOI: 10.1155/2015/979645
16. Holcar M, Goropevšek A, Ihan A, Avčin T. Age-Related Differences in Percentages of Regulatory and Effector T Lymphocytes and Their Subsets in Healthy Individuals and Characteristic STAT1/STAT5 Signalling Response in Helper T Lymphocytes. J Immunol Res. 2015;2015:352934. DOI: 10.1155/2015/352934
17. Frasca D, Diaz A, Romero M, Ferracci F, Blomberg BB. MicroRNAs miR-155 and miR-16 Decrease AID and E47 in B Cells from Elderly Individuals. J Immunol. 2015;195(5):2134‑40. DOI: 10.4049/jimmunol.1500520
18. Tarazona R, Campos C, Pera A, Sanchez-Correa B, Solana R. Flow Cytometry Analysis of NK Cell Phenotype and Function in Aging. Methods Mol Biol. 2015;1343:9‑18. DOI: 10.1007/978-1-4939-2963-4_2
19. Frasca D, Diaz A, Blomberg BB. Activation-Induced Cytidine Deaminase and Switched Memory B Cells as Predictors of Effective In Vivo Responses to the Influenza Vaccine. Methods Mol Biol. 2015;1343:107‑14. DOI: 10.1007/978-1-4939-2963-4_9
20. Jergović M, Tomičević M, Vidović A, Bendelja K, Savić A, Vojvoda V, et al. Telomere shortening and immune activity in war veterans with posttraumatic stress disorder. Prog Neuropsychopharmacol Biol Psychiatry. 2014;54:275‑83. DOI: 10.1016/j.pnpbp.2014.06.010
21. Chou JP, Ramirez CM, Ryba DM, Koduri MP, Effros RB. Prostaglandin E2 Promotes Features of Replicative Senescence in Chronically Activated Human CD8+ T Cells. PLOS ONE. Public Library of Science; 2014;9(6):e99432. DOI: 10.1371/journal.pone.0099432
22. Busse S, Steiner J, Micheel J, Dobrowolny H, Mawrin C, Krause TJ, et al. Age-related increase of VGF-expression in T lymphocytes. Aging (Albany NY). 2014;6(6):440‑53. DOI: 10.18632/aging.100656
23. Compté N, Bailly B, De Breucker S, Goriely S, Pepersack T. Study of the association of total and differential white blood cell counts with geriatric conditions, cardio-vascular diseases, seric IL-6 levels and telomere length. Exp Gerontol. 2015;61:105‑12. DOI: 10.1016/j.exger.2014.11.016
24. Prakash S, Agrawal S, Cao J, Gupta S, Agrawal A. Impaired secretion of interferons by dendritic cells from aged subjects to influenza : role of histone modifications. Age (Dordr). 2013;35(5):1785‑97. DOI: 10.1007/s11357-012-9477-8
25. Álvarez-Rodríguez L, López-Hoyos M, Muñoz-Cacho P, Martínez-Taboada VM. Aging is associated with circulating cytokine dysregulation. Cellular immunology. Elsevier; 2012;273(2):124‑32.
26. Orsini G, Legitimo A, Failli A, Massei F, Biver P, Consolini R. Enumeration of human peripheral blood dendritic cells throughout the life. Int Immunol. 2012;24(6):347‑56. DOI: 10.1093/intimm/dxs006
27. Guerra-Laso JM, González-García S, González-Cortés C, Diez-Tascón C, López-Medrano R, Rivero-Lezcano OM. Macrophages from elders are more permissive to intracellular multiplication of Mycobacterium tuberculosis. Age (Dordr). 2013;35(4):1235‑50. DOI: 10.1007/s11357-012-9451-5
28. Priyanka HP, Sharma U, Gopinath S, Sharma V, Hima L, ThyagaRajan S. Menstrual cycle and reproductive aging alters immune reactivity, NGF expression, antioxidant enzyme activities, and intracellular signaling pathways in the peripheral blood mononuclear cells of healthy women. Brain Behav Immun. 2013;32:131‑43. DOI: 10.1016/j.bbi.2013.03.008
29. Garbe K, Bratke K, Wagner S, Virchow JC, Lommatzsch M. Plasmacytoid dendritic cells and their Toll-like receptor 9 expression selectively decrease with age. Hum Immunol. 2012;73(5):493‑7. DOI: 10.1016/j.humimm.2012.02.007
30. Frasca D, Diaz A, Romero M, Phillips M, Mendez NV, Landin AM, et al. Unique biomarkers for B-cell function predict the serum response to pandemic H1N1 influenza vaccine. Int Immunol. 2012;24(3):175‑82. DOI: 10.1093/intimm/dxr123
31. Longo DM, Louie B, Putta S, Evensen E, Ptacek J, Cordeiro J, et al. Single-cell network profiling of peripheral blood mononuclear cells from healthy donors reveals age- and race-associated differences in immune signaling pathway activation. J Immunol. 2012;188(4):1717‑25. DOI: 10.4049/jimmunol.1102514
32. Hearps AC, Maisa A, Cheng W-J, Angelovich TA, Lichtfuss GF, Palmer CS, et al. HIV infection induces age-related changes to monocytes and innate immune activation in young men that persist despite combination antiretroviral therapy. AIDS. 2012;26(7):843‑53. DOI: 10.1097/QAD.0b013e328351f756
33. Yalcin AD, Gorczynski RM, Kahraman MS, Demirel MU, Terzioglu E. CD40, CD45 CTLA-4 levels are elevated in healthy older adults. Clin Lab. 2012;58(5‑6):449‑56.
34. Ferrando-Martínez S, Ruiz-Mateos E, Hernández A, Gutiérrez E, Rodríguez-Méndez Mdel M, Ordoñez A, Leal M. Age-related deregulation of naive T cell homeostasis in elderly humans. Age (Dordr). 2011 Jun;33(2):197-207. doi: 10.1007/s11357-010-9170-8.
35. Goetzl EJ, Huang M-C, Kon J, Patel K, Schwartz JB, Fast K, et al. Gender specificity of altered human immune cytokine profiles in aging. FASEB J. 2010;24(9):3580‑9. DOI: 10.1096/fj.10-160911
36. Kaszubowska L, Kaczor JJ, Hak L, Dettlaff-Pokora A, Szarynska M, Kmiec Z. Sensitivity of natural killer cells to activation in the process of ageing is related to the oxidative and inflammatory status of the elderly. J Physiol Pharmacol. 2011;62(1):101‑9.
37. Ogden S, Dearman R, Kimber I, Griffiths C. The effect of ageing on phenotype and function of monocyte-derived Langerhans cells. Br J Dermatol. 2011;165(1):184‑8. DOI: 10.1111/j.1365-2133.2011.10313.x
38. Vilas-Boas V, Silva R, Gaio AR, Martins AM, Lima SC, Cordeiro-da-Silva A, et al. P-glycoprotein activity in human Caucasian male lymphocytes does not follow its increased expression during aging. Cytometry A. 2011;79(11):912‑9. DOI: 10.1002/cyto.a.21135
39. Marttila S, Jylhävä J, Pesu M, Hämäläinen S, Jylhä M, Hervonen A, et al. IL-7 concentration is increased in nonagenarians but is not associated with markers of T cell immunosenescence. Exp Gerontol. 2011;46(12):1000‑2. DOI: 10.1016/j.exger.2011.09.004
40. Kimura M, Gazitt Y, Cao X, Zhao X, Lansdorp PM, Aviv A. Synchrony of telomere length among hematopoietic cells. Exp Hematol. 2010;38(10):854‑9. DOI: 10.1016/j.exphem.2010.06.010
41. Horvathova M, Jahnova E, Szabova M, Tulinska M, Kuricova M, Liskova A, et al. The relationship between cell surface markers, cytokines, ageing, and cigarette smoking. Bratisl Lek Listy. 2009;110(7):394‑400.
42. Kushner EJ, Weil BR, MacEneaney OJ, Morgan RG, Mestek ML, Van Guilder GP, et al. Human aging and CD31+ T-cell number, migration, apoptotic susceptibility, and telomere length. J Appl Physiol (1985). 2010;109(6):1756‑61. DOI: 10.1152/japplphysiol.00601.2010
43. Caraux A, Klein B, Paiva B, Bret C, Schmitz A, Fuhler GM, et al. Circulating human B and plasma cells. Age-associated changes in counts and detailed characterization of circulating normal CD138- and CD138+ plasma cells. Haematologica. 2010;95(6):1016‑20. DOI: 10.3324/haematol.2009.018689
44. Panda A, Qian F, Mohanty S, van Duin D, Newman FK, Zhang L, et al. Age-associated decrease in TLR function in primary human dendritic cells predicts influenza vaccine response. J Immunol. 2010;184(5):2518‑27. DOI: 10.4049/jimmunol.0901022
45. Colonna-Romano G, Bulati M, Aquino A, Pellicanò M, Vitello S, Lio D, et al. A double-negative (IgD−CD27−) B cell population is increased in the peripheral blood of elderly people. Mechanisms of Ageing and Development. 2009;130(10):681‑90. DOI: 10.1016/j.mad.2009.08.003
46. Hoffmann J, Erben Y, Zeiher AM, Dimmeler S, Spyridopoulos I. Telomere length-heterogeneity among myeloid cells is a predictor for chronological ageing. Exp Gerontol. 2009;44(5):363‑6. DOI: 10.1016/j.exger.2009.02.006
47. Kilpatrick RD, Rickabaugh T, Hultin LE, Hultin P, Hausner MA, Detels R, et al. Homeostasis of the Naive CD4+ T Cell Compartment during Aging. J Immunol. 2008;180(3):1499‑507.
48. Kaszubowska L, Dettlaff-Pokora A, Hak L, Szarynska M, Ryba M, Mysliwska J, et al. Successful ageing of nonagenarians is related to the sensitivity of NK cells to activation. J Physiol Pharmacol. 2008;59 Suppl 9:187‑99.
49. Simone R, Zicca A, Saverino D. The frequency of regulatory CD3+CD8+CD28- CD25+ T lymphocytes in human peripheral blood increases with age. J Leukoc Biol. 2008;84(6):1454‑61. DOI: 10.1189/jlb.0907627
50. Ghia P, Prato G, Stella S, Scielzo C, Geuna M, Caligaris-Cappio F. Age-dependent accumulation of monoclonal CD4+CD8+ double positive T lymphocytes in the peripheral blood of the elderly. Br J Haematol. 2007;139(5):780‑90. DOI: 10.1111/j.1365-2141.2007.06867.x
51. Agrawal A, Agrawal S, Cao J-N, Su H, Osann K, Gupta S. Altered Innate Immune Functioning of Dendritic Cells in Elderly Humans: A Role of Phosphoinositide 3-Kinase-Signaling Pathway. The Journal of Immunology. American Association of Immunologists; 2007;178(11):6912‑22. DOI: 10.4049/jimmunol.178.11.6912
52. Mazzatti DJ, White A, Forsey RJ, Powell JR, Pawelec G. Gene expression changes in long-term culture of T-cell clones: genomic effects of chronic antigenic stress in aging and immunosenescence. Aging Cell. 2007;6(2):155‑63. DOI: 10.1111/j.1474-9726.2007.00269.x
53. Njemini R, Lambert M, Demanet C, Mets T. The effect of aging and inflammation on heat shock protein 27 in human monocytes and lymphocytes. Exp Gerontol. 2006;41(3):312‑9. DOI: 10.1016/j.exger.2006.01.006
54. Walrand S, Guillet C, Boirie Y, Vasson M-P. Insulin differentially regulates monocyte and polymorphonuclear neutrophil functions in healthy young and elderly humans. J Clin Endocrinol Metab. 2006;91(7):2738‑48. DOI: 10.1210/jc.2005-1619
55. Ju Y-J, Lee K-H, Park J-E, Yi Y-S, Yun M-Y, Ham Y-H, et al. Decreased expression of DNA repair proteins Ku70 and Mre11 is associated with aging and may contribute to the cellular senescence. Experimental & molecular medicine. Nature Publishing Group; 2006;38(6):686‑93.
56. Kovaiou RD, Weiskirchner I, Keller M, Pfister G, Cioca DP, Grubeck-Loebenstein B. Age-related differences in phenotype and function of CD4+ T cells are due to a phenotypic shift from naive to memory effector CD4+ T cells. Int Immunol. 2005;17(10):1359‑66. DOI: 10.1093/intimm/dxh314
57. Poggioli S, Mary J, Bakala H, Friguet B. Evidence of preferential protein targets for age-related modifications in peripheral blood lymphocytes. Ann N Y Acad Sci. 2004;1019:211‑4. DOI: 10.1196/annals.1297.034
58. Larbi A, Dupuis G, Douziech N, Khalil A, Fülöp T. Low-grade inflammation with aging has consequences for T-lymphocyte signaling. Ann N Y Acad Sci. 2004;1030:125‑33. DOI: 10.1196/annals.1329.016
59. Simar D, Malatesta D, Koechlin C, Cristol JP, Vendrell JP, Caillaud C. Effect of age on Hsp72 expression in leukocytes of healthy active people. Exp Gerontol. 2004;39(10):1467‑74. DOI: 10.1016/j.exger.2004.08.002
60. Son NH, Joyce B, Hieatt A, Chrest FJ, Yanovski J, Weng N. Stable telomere length and telomerase expression from naïve to memory B-lymphocyte differentiation. Mech Ageing Dev. 2003;124(4):427‑32. DOI: 10.1016/s0047-6374(03)00018-6
61. Breitbart E, Wang X, Leka LS, Dallal GE, Meydani SN, Stollar BD. Altered memory B-cell homeostasis in human aging. J Gerontol A Biol Sci Med Sci. 2002;57(8):B304-311. DOI: 10.1093/gerona/57.8.b304
62. Tsaknaridis L, Spencer L, Culbertson N, Hicks K, LaTocha D, Chou YK, et al. Functional assay for human CD4+CD25+ Treg cells reveals an age-dependent loss of suppressive activity. J Neurosci Res. 2003;74(2):296‑308. DOI: 10.1002/jnr.10766
63. Kimmig S, Przybylski GK, Schmidt CA, Laurisch K, Möwes B, Radbruch A, et al. Two Subsets of Naive T Helper Cells with Distinct T Cell Receptor Excision Circle Content in Human Adult Peripheral Blood. J Exp Med. 2002;195(6):789‑94. DOI: 10.1084/jem.20011756
64. Njemini R, Abeele MV, Demanet C, Lambert M, Vandebosch S, Mets T. Age-related decrease in the inducibility of heat-shock protein 70 in human peripheral blood mononuclear cells. J Clin Immunol. 2002;22(4):195‑205. DOI: 10.1023/a:1016036724386
65. Poggioli S, Bakala H, Friguet B. Age-related increase of protein glycation in peripheral blood lymphocytes is restricted to preferential target proteins. Exp Gerontol. 2002;37(10‑11):1207‑15. DOI: 10.1016/s0531-5565(02)00145-6
66. McNerlan SE, Rea IM, Alexander HD. A whole blood method for measurement of intracellular TNF-alpha, IFN-gamma and IL-2 expression in stimulated CD3+ lymphocytes: differences between young and elderly subjects. Exp Gerontol. 2002;37(2‑3):227‑34. DOI: 10.1016/s0531-5565(01)00188-7
67. Olaussen RW, Farstad IN, Brandtzaeg P, Rugtveit J. Age-related changes in CCR9+ circulating lymphocytes: are CCR9+ naive T cells recent thymic emigrants? Scand J Immunol. 2001;54(5):435‑9. DOI: 10.1046/j.1365-3083.2001.01008.x
68. Ginaldi L, De Martinis M, Modesti M, Loreto F, Corsi MP, Quaglino D. Immunophenotypical changes of T lymphocytes in the elderly. Gerontology. 2000;46(5):242‑8. DOI: 10.1159/000022167
69. De Martinis M, Modesti M, Loreto MF, Quaglino D, Ginaldi L. Adhesion molecules on peripheral blood lymphocyte subpopulations in the elderly. Life Sci. 2000;68(2):139‑51. DOI: 10.1016/s0024-3205(00)00924-3
70. De Martinis M, Modesti M, Profeta VF, Tullio M, Loreto MF, Ginaldi L, et al. CD50 and CD62L adhesion receptor expression on naive (CD45RA+) and memory (CD45RO+) T lymphocytes in the elderly. Pathobiology. 2000;68(6):245‑50. DOI: 10.1159/000055933
71. Myśliwska J, Bryl E, Trzonkowski P, Myśliwski A. Compensatory effect of TNFalpha on low natural killer activity in the elderly. Acta Biochim Pol. 2000;47(2):301‑11.
72. Yen CJ, Lin SL, Huang KT, Lin RH. Age-associated changes in interferon-gamma and interleukin-4 secretion by purified human CD4+ and CD8+ T cells. J Biomed Sci. 2000;7(4):317‑21. DOI: 10.1007/bf02253251
73. Beinert T, Münzing S, Possinger K, Krombach F. Increased expression of the tetraspanins CD53 and CD63 on apoptotic human neutrophils. Journal of Leukocyte Biology. 2000;67(3):369‑73. DOI: 10.1002/jlb.67.3.369
74. Pietschmann P, Hahn P, Kudlacek S, Thomas R, Peterlik M. Surface markers and transendothelial migration of dendritic cells from elderly subjects. Exp Gerontol. 2000;35(2):213‑24. DOI: 10.1016/s0531-5565(99)00089-3
