# Supplementary material for: Immunosenescence Study of T Cells: A Systematic Review
Source: Front Immunol. 2021 Jan 15;11:604591. doi: 10.3389/fimmu.2020.604591 (PMC7843425; doi:10.3389/fimmu.2020.604591)
Supplement: Supplementary file 4 [file Table_4.docx]

**Table S4. Summary of published studies considered in the review.**

| Reference | # | Date of publication | Lymphocyte definition | Elderly | Young | Memory definition | Biomarkers in elderly | Cytokines | Telomere length | CMV serology | Stimulation |
| --- | --- | --- | --- | --- | --- | --- | --- | --- | --- | --- | --- |
|  |  |  |  | n | n |  |  |  |  |  |  |
| Xu, et al. | 1 | 2019 | γ/δ | 24 | 22 | Yes | Yes | Yes | Yes | Yes | No |
| Ross et al. | 2 | 2018 | CD4/CD8 | 10 | 9 | No | Yes | No | No | No | No |
| Sizzano et al. | 3 | 2018 | CD4/CD8 | 7 | 7 | Yes | No | No | No | No | No |
| Bajwa et al. | 4 | 2017 | CD4/CD8 | 103 | 55 | Yes | No | No | No | Yes | No |
| Onyema et al. | 5 | 2015 | CD8 | 22 | 11 | No | Yes | No | No | No | No |
| Riddell et al. | 6 | 2015 | CD8 | 125 | | Yes | No | Yes | Yes | Yes | No |
| Henson et al. | 7 | 2015 | CD8 | 31 | 33 | Yes | Yes | Yes | No | No | Yes |
| Britanova et al. | 8 | 2014 | CD4/CD8 | 7 | 10 | Yes | No | No | No | No | No |
| Larbi et al. | 9 | 2014 | CD4/CD8 | 15 | 15 | Yes | No | Yes | No | No | No |
| Canaday et al. | 10 | 2013 | CD4/CD8 | 24 | 24 | No | Yes | No | No | No | Yes |
| Dolfi et al. | 11 | 2013 | CD8 | 405 | 207 | Yes | Yes | Yes | No | No | No |
| Lee et al. | 12 | 2012 | CD8 | 43 | 62 | Yes | No | No | No | Yes | No |
| Onyema et al. | 13 | 2012 | CD8 | 11 | 11 | No | Yes | No | No | Yes | No |
| Libri et al. | 14 | 2011 | CD4 | 67 | 40 | Yes | Yes | No | No | Yes | No |
| Agius et al. | 15 | 2009 | CD4 | 81 | 81 | No | Yes | Yes | No | No | No |
| Herndler-Brandstetter et al. | 16 | 2008 | CD8 | NR | NR | No | Yes | No | Yes | No | No |
| Czesnikiewicz-Guzik et al. | 17 | 2008 | CD47CD8 | 41 | 68 | Yes | Yes | No | No | No | No |
| Nasi et al. | 18 | 2006 | CD4/CD8 | 44 | 29 | Yes | Yes | No | No | No | No |
| Sawhney et al. | 19 | 2006 | CD4/CD8 | 25 | 20 | No | Yes | No | No | No | Yes |
| Alberti et al. | 20 | 2006 | CD4 | 20 | 12 | Yes | No | Yes | No | No | No |
| He et al. | 21 | 2006 | CD8 | 15 | 22 | No | Yes | No | No | Yes | No |
| Pinti et al. | 22 | 2004 | CD4/CD8 | 14 | 13 | No | Yes | No | No | No | No |
| Hong et al. | 23 | 2004 | CD8 | 17 | 17 | Yes | No | No | No | No | No |
| Deng et al. | 24 | 2004 | CD4/CD8 | 11 | 5 | No | No | Yes | No | No | Yes |
| Mariani et al. | 25 | 2003 | CD8 | 19 | 18 | No | No | No | Yes | No | No |
| Ouyang et al. | 26 | 2003 | CD8 | 19 | 10 | No | Yes | Yes | No | Yes | Yes |
| Machado et al. | 27 | 2003 | CD4/CD8 | 10 | 10 | No | Yes | No | No | No | No |
| Ouyang et al. | 28 | 2003 | CD8 | 70 | 11 | No | Yes | Yes | No | Yes | Yes |
| Pietschmann et al. | 29 | 2003 | CD4/CD8 | 79 | 75 | No | No | Yes | No | No | Yes |
| Trzonkowski et al. | 30 | 2003 | CD8 | 91 | 63 | No | Yes | Yes | No | Yes | Yes |
| Zanni et al. | 31 | 2003 | CD8 | 10 | 10 | Yes | No | Yes | No | No | No |
| Sandmand et al. | 32 | 2003 | CD4/CD8 | 14 | 25 | No | No | Yes | No | No | Yes |
| Trzonkowski et al. | 33 | 2002 | CD8 | 65 | 31 | No | Yes | No | No | No | No |
| Sandmand et al. | 34 | 2002 | CD4/CD8 | 22 | 28 | No | Yes | Yes | No | No | No |
| Eylar et al. | 35 | 2001 | CD4/CD8 | 40 | 48 | No | No | Yes | No | No | Yes |
| Son et al. | 36 | 2000 | CD4/CD8 | 30 | 22 | No | No | No | Yes | No | No |

**References**

1. Xu W, Monaco G, Wong EH, Tan WLW, Kared H, Simoni Y, et al. Mapping of γ/δ T Cells Reveals Vδ2+ T Cells Resistance to Senescence. EBiomedicine 39;44. 2019. PubMed PMID: 30528453.
2. Ross M, Ingram L, Taylor G, Malone E, Simpson RJ, West D, Florida-James G. Older Men Display Elevated Levels of Senescence-Associated Exercise-Responsive CD28 Null Angiogenic T Cells Compared With Younger Men. Physiological reports 6 (12);e13697. 2018. PubMed PMID: 29939490.
3. Sizzano F, Collino S, Cominetti O, Monti D, Garagnani P, Ostan R, et al. Evaluation of Lymphocyte Response to the Induced Oxidative Stress in a Cohort of Ageing Subjects, Including Semisupercentenarians and Their Offspring. Mediators of inflammation 2018;7109312. 2018 2018/02/19.
4. Bajwa M, Vita S, Vescovini R, Larsen M, Sansoni P, Terrazzini N, et al. CMV-Specific T-Cell Responses at Older Ages: Broad Responses With a Large Central Memory Component May Be Key to Long-Term Survival. The journal of infectious diseases 215 (8);1212. 2017 04/15/2017. PubMed PMID: 28199648.
5. Onyema OO, Njemini R, Forti LN, Bautmans I, Aerts JL, De Waele M, Mets T. Aging-Associated Subpopulations of Human CD8+ T-Lymphocytes Identified by Their CD28 and CD57 Phenotypes. Archives of gerontology and geriatrics 61 (3);494. 2015 Nov–Dec. PubMed PMID: 26277688.
6. Riddell NE, Griffiths SJ, Rivino L, King DC, Teo GH, Henson SM, et al. Multifunctional Cytomegalovirus (CMV)-Specific CD8(+) T Cells Are Not Restricted by Telomere-Related Senescence in Young or Old Adults. Immunology 144 (4);549. 2015. PubMed PMID: 25314332.
7. Henson SM, Macaulay R, Riddell NE, Nunn CJ, Akbar AN. Blockade of PD-1 or p38 MAP Kinase Signaling Enhances Senescent Human CD8(+) T-Cell Proliferation by Distinct Pathways. European journal of immunology 45 (5);1441. 2015. PubMed PMID: 25707450.
8. Britanova OV, Putintseva EV, Shugay M, Merzlyak EM, Turchaninova MA, Staroverov DB, et al. Age-Related Decrease in TCR Repertoire Diversity Measured With Deep and Normalized Sequence Profiling. Journal of immunology 192 (6);2689. 2014 03/15/2014. PubMed PMID: 24510963 (Baltimore Md, Putintseva EV, Shugay M, Merzlyak EM, Turchaninova MA, Staroverov DB et al.. Journal of immunology 192 (6);2689 2014).
9. Larbi A, Fortin C, Dupuis G, Berrougui H, Khalil A, Fulop T. Immunomodulatory Role of High-Density Lipoproteins: Impact on Immunosenescence. Age (Dordrecht, Netherlands) 36 (5);9712. 2014. PubMed PMID: 25216565.
10. Canaday DH, Parker KE, Aung H, Chen HE, Nunez-Medina D, Burant CJ. Age-Dependent Changes in the Expression of Regulatory Cell Surface Ligands in Activated Human T-Cells. BMC immunology 14;45. 2013 10/01/2013. PubMed PMID: 24083425.
11. Dolfi DV, Mansfield KD, Polley AM, Doyle SA, Freeman GJ, Pircher H, et al. Increased T-Bet Is Associated With Senescence of Influenza Virus-Specific CD8 T Cells in Aged Humans. Journal of leukocyte biology 93 (6);825. 2013. PubMed PMID: 23440501.
12. Lee WW, Shin MS, Kang Y, Lee N, Jeon S, Kang I. The Relationship of Cytomegalovirus (CMV) Infection With Circulatory IFN-α Levels and IL-7 Receptor α Expression on CD8+ T Cells in Human Aging. Cytokine 58 (3);332. 2012. PubMed PMID: 22484243.
13. Onyema OO, Njemini R, Bautmans I, Renmans W, De Waele M, Mets T. Cellular Aging and Senescence Characteristics of Human T-Lymphocytes. Biogerontology 13 (2);169. 2012 Apr. PubMed PMID: 22102004. Epub 2011/11/22.
14. Libri V, Azevedo RI, Jackson SE, Di Mitri D, Lachmann R, Fuhrmann S, et al. Cytomegalovirus Infection Induces the Accumulation of Short-Lived, Multifunctional CD4+CD45RA+CD27+ T Cells: The Potential Involvement of Interleukin-7 in This Process. Immunology 132 (3);326. 2011. PubMed PMID: 21214539.
15. Agius E, Lacy KE, Vukmanovic-Stejic M, Jagger AL, Papageorgiou AP, Hall S, et al. Decreased TNF-alpha Synthesis by Macrophages Restricts Cutaneous Immunosurveillance by Memory CD4+ T Cells During Aging. The journal of experimental medicine 206 (9);1929. 2009 08/31/2009. PubMed PMID: 19667063.
16. Herndler-Brandstetter D, Veel E, Laschober GT, Pfister G, Brunner S, Walcher S, et al. Non-Regulatory CD8+CD45RO+CD25+ T-Lymphocytes May Compensate for the Loss of Antigen-Inexperienced CD8+CD45RA+ T-Cells in Old Age. Biological chemistry 389 (5);561. 2008. PubMed PMID: 18953723.
17. Czesnikiewicz-Guzik M, Lee WW, Cui D, Hiruma Y, Lamar DL, Yang ZZ, et al. T Cell Subset-Specific Susceptibility to Aging. Clinical immunology 127 (1);107. 2008. PubMed PMID: 18222733.
18. Nasi M, Troiano L, Lugli E, Pinti M, Ferraresi R, Monterastelli E, et al. Thymic Output and Functionality of the IL-7/IL-7 Receptor System in Centenarians: Implications for the Neolymphogenesis at the Limit of Human Life. Aging cell 5 (2);167. 2006. PubMed PMID: 16626395.
19. Sawhney M, Mathew M, Valarmathi MT, Das SN. Age Related Changes in Fas (CD95) and Fas Ligand Gene Expression and Cytokine Profiles in Healthy Indians. Asian Pacific journal of allergy and immunology 24 (1);47. 2006. PubMed PMID: 16913188.
20. Alberti S, Cevenini E, Ostan R, Capri M, Salvioli S, Bucci L, et al. Age-Dependent Modifications of Type 1 and Type 2 Cytokines Within Virgin and Memory CD4+ T Cells in Humans. Mechanisms of ageing and development 127 (6);560. 2006. PubMed PMID: 16516272.
21. He XH, Zha QB, Liu Y, Xu LH, Chi XY. High Frequencies Cytomegalovirus pp65(495-503)-Specific CD8+ T Cells in Healthy Young and Elderly Chinese Donors: Characterization of Their Phenotypes and TCR Vbeta Usage. Journal of clinical immunology 26 (5);417. 2006. PubMed PMID: 16850281.
22. Pinti M, Troiano L, Nasi M, Bellodi C, Ferraresi R, Mussi C, et al. Balanced Regulation of mRNA Production for Fas and Fas Ligand in Lymphocytes From Centenarians: How the Immune System Starts Its Second Century. Circulation 110 (19);3108. 2004 11/09/2004. PubMed PMID: 15505087.
23. Hong MS, Dan JM, Choi JY, Kang I. Age-Associated Changes in the Frequency of Naïve, Memory and Effector CD8+ T Cells. Mechanisms of ageing and development 125 (9);615. 2004. PubMed PMID: 15491679.
24. Deng Y, Jing Y, Campbell AE, Gravenstein S. Age-Related Impaired Type 1 T Cell Responses to Influenza: Reduced Activation Ex Vivo, Decreased Expansion in CTL Culture In Vitro, and Blunted Response to Influenza Vaccination In Vivo in the Elderly. Journal of immunology 172 (6);3437. 2004 03/15/2004. PubMed PMID: 15004143 (Baltimore Md, Jing Y, Campbell AE, Gravenstein S. Journal of immunology 172 (6);3437. 2004).
25. Mariani E, Meneghetti A, Formentini I, Neri S, Cattini L, Ravaglia G, et al. Different Rates of Telomere Shortening and Telomerase Activity Reduction in CD8 T and CD16 NK Lymphocytes With Ageing. Experimental gerontology 38 (6);653. 2003. PubMed PMID: 12814800.
26. Ouyang Q, Wagner WM, Wikby A, Walter S, Aubert G, Dodi AI, et al. Large Numbers of Dysfunctional CD8+ T Lymphocytes Bearing Receptors for a Single Dominant CMV Epitope in the Very Old. Journal of clinical immunology 23 (4);247. 2003. PubMed PMID: 12959217.
27. Machado CG, Calado RT, Garcia AB, Falcão RP. Age-Related Changes of the Multidrug Resistance P-Glycoprotein Function in Normal Human Peripheral Blood T Lymphocytes. Brazilian journal of medical and biological research = revista brasileira de pesquisas medicas e biologicas 36 (12);1653. 2003. PubMed PMID: 14666249.
28. Ouyang Q, Wagner WM, Voehringer D, Wikby A, Klatt T, Walter S, et al. Age-Associated Accumulation of CMV-Specific CD8+ T Cells Expressing the Inhibitory Killer Cell Lectin-Like Receptor G1 (KLRG1). Experimental gerontology 38 (8);911. 2003. PubMed PMID: 12915213.
29. Pietschmann P, Gollob E, Brosch S, Hahn P, Kudlacek S, Willheim M, et al. The Effect of Age and Gender on Cytokine Production by Human Peripheral Blood Mononuclear Cells and Markers of Bone Metabolism. Experimental gerontology 38 (10);1119. 2003. PubMed PMID: 14580865.
30. Trzonkowski P, Myśliwska J, Szmit E, Wieckiewicz J, Lukaszuk K, Brydak LB, et al. Association Between Cytomegalovirus Infection, Enhanced Proinflammatory Response and Low Level of Anti-Hemagglutinins During the Anti-Influenza Vaccination--An Impact of Immunosenescence. Vaccine 21 (25-26);3826. 2003 09/08/2003. PubMed PMID: 12922116.
31. Zanni F, Vescovini R, Biasini C, Fagnoni F, Zanlari L, Telera A, et al. Marked Increase With Age of Type 1 Cytokines Within Memory and Effector/Cytotoxic CD8+ T Cells in Humans: A Contribution to Understand the Relationship Between Inflammation and Immunosenescence. Experimental gerontology 38 (9);981. 2003. PubMed PMID: 12954485.
32. Sandmand M, Bruunsgaard H, Kemp K, Andersen-Ranberg K, Schroll M, Jeune B. High Circulating Levels of Tumor Necrosis Factor-Alpha in Centenarians Are Not Associated With Increased Production in T Lymphocytes. Gerontology 49 (3);155. 2003 May–Jun. PubMed PMID: 12679605.
33. Trzonkowski P, Myśliwska J, Szmit E, Zak M, Foerster J, Myśliwski A. Lower Percentage of CD8(High+)CD152(+) but Not CD8(High+)CD28(+) T Lymphocytes in the Elderly May Be Reverted by Interleukin 2 In Vitro. Mechanisms of ageing and development 123 (9);1283. 2002. PubMed PMID: 12020949.
34. Sandmand M, Bruunsgaard H, Kemp K, Andersen-Ranberg K, Pedersen AN, Skinhøj P, Pedersen BK. Is Ageing Associated With a Shift in the Balance Between Type 1 and Type 2 Cytokines in Humans? Clinical and experimental immunology 127 (1);107. 2002. PubMed PMID: 11882040.
35. Eylar EH, Lefranc CE, Yamamura Y, Báez I, Colón-Martinez SL, Rodriguez N, Breithaupt TB. HIV Infection and Aging: Enhanced Interferon- and Tumor Necrosis Factor-Alpha Production by the CD8+ CD28- T Subset. BMC immunology 2;10. 2001. PubMed PMID: 11696237.
36. Son NH, Murray S, Yanovski J, Hodes RJ, Weng N. Lineage-Specific Telomere Shortening and Unaltered Capacity for Telomerase Expression in Human T and B Lymphocytes With Age. Journal of immunology 165 (3);1191. 2000 08/01/2000. PubMed PMID: 10903716 (Baltimore Md, Murray S, Yanovski J, Hodes RJ, Weng N. Journal of immunology 165 (3);1191. 2000).
